# Universal Kinetics of the Onset of Cell Spreading on Substrates of Different Stiffness

**DOI:** 10.1016/j.bpj.2018.12.020

**Published:** 2019-01-05

**Authors:** Samuel Bell, Anna-Lena Redmann, Eugene M. Terentjev

**Affiliations:** 1 Cavendish Laboratory, University of Cambridge, Cambridge, United Kingdom

## Abstract

When plated onto substrates, cell morphology and even stem-cell differentiation are influenced by the stiffness of their environment. Stiffer substrates give strongly spread (eventually polarized) cells with strong focal adhesions and stress fibers; very soft substrates give a less developed cytoskeleton and much lower cell spreading. The kinetics of this process of cell spreading is studied extensively, and important universal relationships are established on how the cell area grows with time. Here, we study the population dynamics of spreading cells, investigating the characteristic processes involved in the cell response to the substrate. We show that unlike the individual cell morphology, this population dynamics does not depend on the substrate stiffness. Instead, a strong activation temperature dependence is observed. Different cell lines on different substrates all have long-time statistics controlled by the thermal activation over a single energy barrier *ΔG* ≈ 18 kcal/mol, whereas the early-time kinetics follows a power law ∼*t*^5^. This implies that the rate of spreading depends on an internal process of adhesion complex assembly and activation; the operational complex must have five component proteins, and the last process in the sequence (which we believe is the activation of focal adhesion kinase) is controlled by the binding energy *ΔG*.

## Introduction

Matrix stiffness is known to affect cell size and morphology (1, 2, 3). When cells are plated onto soft substrates, their footprint will not increase as much as on stiff substrates, and their spreading will be more isotropic; the resulting cells will be round and dome-like in shape. On stiff substrates, the same cells will spread very strongly, develop concentrated focal adhesion clusters and stress fibers of bundled F-actin, and eventually polarize to initiate migration. This leads to several well-documented biological functions in tissues: variable stem-cell differentiation pathways (1, 4), the fibroblast-myofibroblast transition near scar tissue (5, 6, 7), fibrosis in smooth-muscle cells near rigid plaque or scar tissue (8, 9), and the stiffer nature of tumor cells (10, 11). The definitive review (12) summarizes this topic.

The actual process of spreading, after a planktonic cell is deposited on a substrate, involves several stages. After initial anchoring, which probably occurs because of a nonspecific hydrophobic or van der Waals binding, one could see an initial increase of the cell footprint on the surface because of viscoelastic wetting (13, 14). Once on the surface, the cell must test for the presence of suitable ligands and then bind to them (15, 16). This specific adhesion must occur for the cell to spread (17). Then, the cell tests the elasticity of the extracellular matrix (ECM), and on sufficiently stiff substrates, it continues spreading, approaching its maximal footprint area. Finally, after polarization is triggered on stiff substrates, the cell may start moving in a particular direction.

The dynamics of cells spreading has been studied extensively, and several characteristic universal features have been established (2, 12, 18, 19, 20). In particular, the average cell area has been shown to grow with time as a power law, often with the radius of cell footprint being *R* ∝ [*t* − *τ*_lag_]^1/2^, where the “lag” *τ*_lag_ is referred to as the adhesion time (18, 21, 22, 23). It is important to note that the “lag time” is observed in many discussions of the dynamics of spreading but mostly ignored by subtracting it from the data. Several mechanistic models have been developed of how the cell spreading is achieved after the adhesion to ECM is established (18, 21, 23) as well as the spreading and cell orientation response to mechanical deformation of the substrate (24, 25). A common theme to these studies is the presentation of individual cell trajectories, outlining the time course of a cell response to adhesion (although, of course, many cells are used to generate statistics). In contrast, here, we examine the dynamics of a cell population by identifying the time at which a cell reaches a specific point early in its spreading sequence (essentially reflecting the “decision” of a cell to start spreading in response to its ECM mechanosensing signal). Frisch and Thoumine (26) have shown that in the early stages of spreading, the cell takes a spherical-cap morphology, and when the increasing adhesion energy becomes similar to the cell cortical tension, the cell contact angle crosses from greater than 90° (representing the partial dewetting) to less than 90° (representing the partial wetting). Such a binary condition, asking whether an event has taken place by a certain time rather than what events are taking place over the course of time, allows the use of stochastic theory to interrogate the cell dynamics, extracting useful information about the underlying kinetics of spreading. In particular, we are able to form a better understanding of the “lag time” and also identify the rate-limiting energy barrier that controls the transition of cells from the initial nonspecific binding to the final strongly adhered and widely spreading regime. This is a useful complementary approach to single-cell measurements. We also emphasize that here, and in the rest of this article, we are discussing isolated cells on a substrate; when cells adhere to each other, their shape transitions are controlled by other mechanisms, based on cadherin and associated pathways (27).

**Figure 1:**
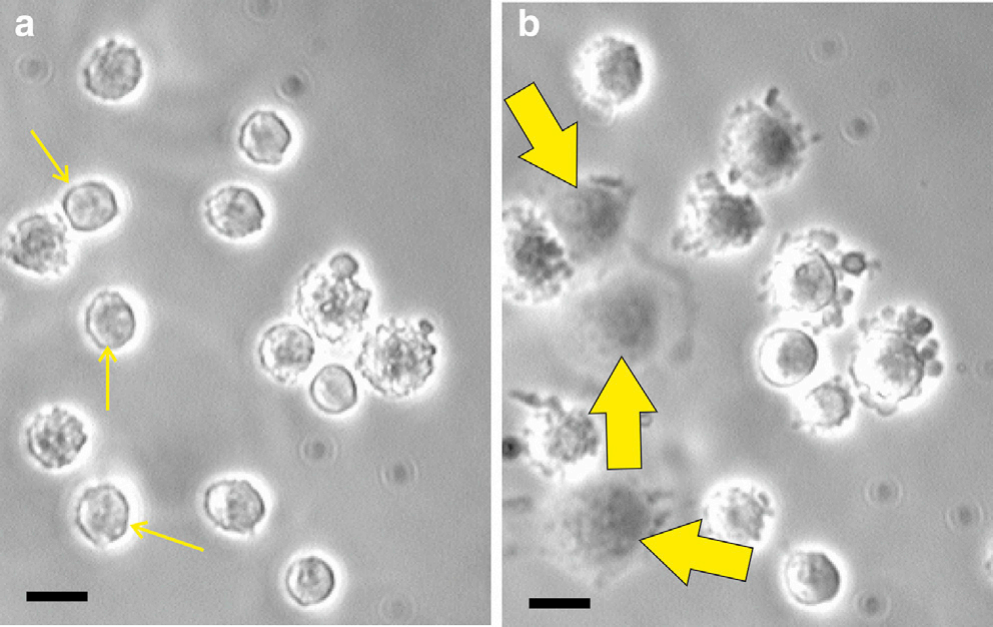
A section of the experimental field of view, illustrating the onset of spreading. Photographs (*a*) and (*b*) show the same cells immediately after planting on the substrate (*solid glass* with fibronectin) and 15 min later, when several cells have already responded by spreading (labeled by *matching arrows*). Scale bars, 20 *μ*m. To see this figure in color, go online.

**Figure 2:**
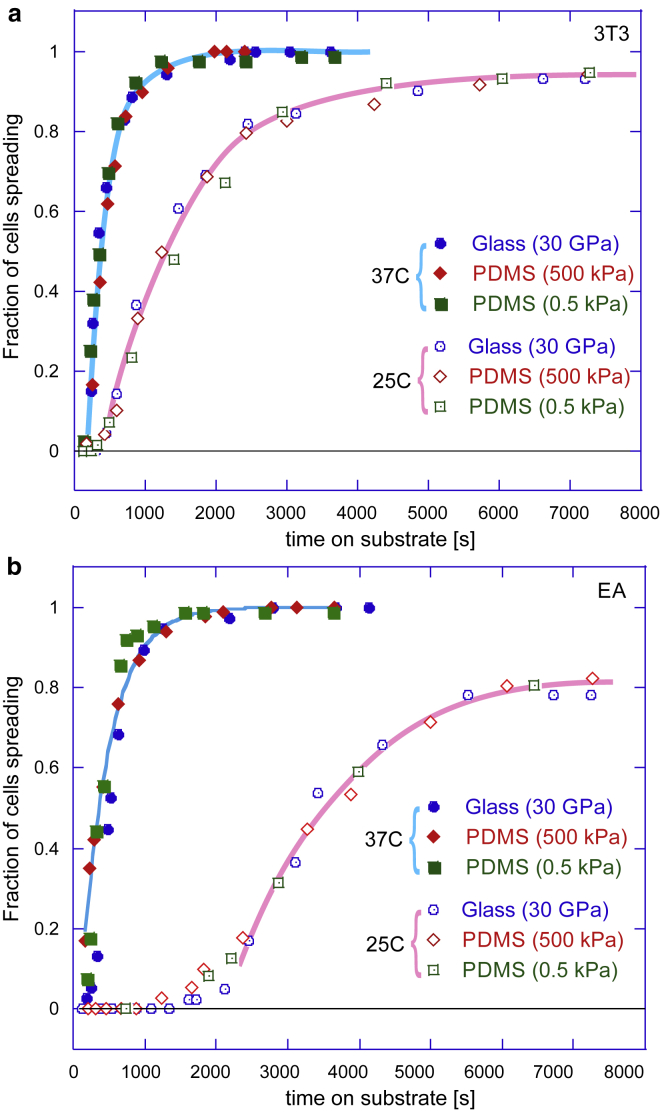
Cumulative population dynamics of cell spreading. Plots (*a*) and (*b*) show the growing fraction of cells engaged in spreading on substrates with different stiffness for 3T3 fibroblasts and EA endothelial cells at two different temperatures each. It is clear that the dynamics is not affected by the substrate stiffness but is affected by changes with temperature. In the remainder of this article, we analyze in detail the long-time behavior of these cumulative curves as they approach saturation, and the behavior at short times when the onset of mechanosensing response occurs. To see this figure in color, go online.

While reporting and discussing the cell area increase on stiffer substrates, Fig. 5d of the article by Yeung et al. (2) and Fig. 2A of the article by Reinhart-King et al. (20) also present data on the time dependence of cell spreading, which already gives a hint for our central experimental finding: the onset of cell spreading does not depend on the substrate. In this article, we investigate the time dependence (kinetics) of the initiation of spreading, asking the following question: how long does it take for the cell to recognize the presence of a substrate and respond by engaging signaling pathways and enacting the required morphological change (spreading on the substrate)? Fig. 1 illustrates the point: plots (Fig. 1, *a* and *b*) show the same cells immediately after planting on the substrate and after some time when several cells have already responded by engaging their spreading. We plated two very different cell lines (National Institutes of Health (NIH)/3T3 fibroblasts and EA.hy927 endothelial cells) on a variety of substrates that span the range of stiffness from 30 GPa (stiff glass) to 460 Pa (very soft gel), registering the characteristic time at which the initially deposited planktonic cells start to spread.

We discover three things: 1) the onset of spreading is completely universal, not depending on the stiffness of substrates (in contrast to the final cell morphology, which strongly depends on it); 2) the rate-limiting process, with the characteristic free energy barrier, is the same in both cell lines; and 3) the onset of spreading is controlled by a nucleation event, its universal power-law dependence *t*^5^, suggesting that there are five state changes a newly deposited cell must go through before it is able to spread. We also measure the sum of the free-energy changes of these state changes and find that this, in contrast to the rate-limiting process, depends on the cell line.

At first, our results on the insensitivity of the onset of spreading to substrate stiffness look counter to much of the literature. It is important to draw a clear line between many existing results on the cell area increase with time on different substrates, and our study looking at the statistics of a cell population that is starting to spread. In particular, the criterion we observe happens at a very early stage of the overall spreading (see Fig. S3), in which the cell area has increased only by a factor of 1.26 from its initial settled state.

## Materials and Methods

### Cells and cell culture procedures

We chose to study endothelial cells and fibroblasts because their adhesion behavior is important for understanding cardiovascular diseases and tissue engineering, using immortalized cell lines: NIH/3T3 murine fibroblasts (obtained from American Type Culture Collection, Manassas, VA) and EA.hy927 endothelial cells.

NIH/3T3 fibroblasts are very well characterized because they have been used in many cell studies since their establishment as a cell line; they have also been used in cell adhesion studies, making them a good choice for our experiments (28, 29). EA.hy927 is a cell line established in 1983 by the fusion of human umbilical vein endothelial cells with a lung carcinoma line (30). It has since become a widely used and thus well-characterized cell line, popular in studies of cardiovascular diseases. EA.hy927 cells have also been used for adhesion strength assays (31).

Cells were normally cultured at 37°C and 5% CO_2_ in Dulbecco’s modified Eagle’s medium, from Greiner (Monroe, NC), with 10% fetal bovine serum and 1% penicillin/streptomycin, from Sigma-Aldrich (St. Louis, MO) (see Supporting Materials and Methods for detail). For a comparative study of the role of nutrients in the medium, we also used phosphate-buffered saline (PBS) from Thermo Fisher Scientific (Waltham, MA) during the spreading experiments.

### Substrates of varying stiffness

To span a wide range of substrate stiffness, we used standard laboratory glass (elastic modulus 30 GPa) and several versions of siloxane elastomers: Sylgard 184 and Sylgard 527, the latter used with the compound/hardener ratio of 1:1 and 5:4. The resulting elastomers were tested on a standard laboratory rheometer (Anton Paar, Graz, Austria), giving the values of equilibrium modulus *G* = 460 Pa (for Syl527 5:4), 480 kPa (for Syl184), and 30 GPa for glass (zero-frequency limit shown in Fig. S1). For comparison, the stiffness of typical mammalian tissues is 100 Pa–1 kPa in brain tissue, ∼3 kPa in adipose tissue, 10–20 kPa in muscle, 30–50 kPa in fibrous tissue, and up to a few MPa for bone. We avoided applying the commonly used plasma treatment because this was making the surface highly uneven on a micron scale, which would affect the adhesion. All surfaces were cleaned by ultrasonication in 96% ethanol for 15 min and then incubated with 10 *μ*g/mL fibronectin in PBS for 45 min.

### Experimental procedure and data acquisition

In our standard cell-spreading experiment, the cell culture dish was inserted into a closed chamber that maintained controlled temperature with an active water bath, and the CO_2_ atmosphere, with microscope observation from the top. The cell culture (density 5 × 10^5^ cells per mL, counted by the Neubauer chamber) was placed over the entire substrate. Cells were left to adhere to the substrate for 2 min, at which point the culture dish containing the substrate was filled slowly with fresh medium to reduce the cell density. This was to prevent new cells depositing and cell clusters forming on the substrate. Only the cells attached to the substrate at this point were included in the subsequent counting. This initial attachment is certainly purely physical through van der Waals forces and various nonspecific cell adhesion molecule headgroups. These physically adhered cells, initially spherical in planktonic culture, maintain the high spherical-cap shape with only a small adhesion footprint as ordinary inflated bilayer vesicles would do as well. This is readily confirmed by the optical interference bands around the cell perimeter and the lensing effect focusing the light by the short-focal distance near-spherical shape (see Supporting Materials and Methods, and also (26) for detail).

After a certain time on the substrate, the cells finally engage their specific adhesion-mechanosensing mechanism and start spreading, achieving a very widely spread area with highly asymmetric focal adhesions on stiff substrates or a round dome-like shape on soft substrates. We are looking to determine the time it takes for the cells to engage this active spreading process.

To obtain a population distribution of the onset time of cell spreading, we had to choose a spreading criterion that would be clear and easily distinguishable to avoid counting errors. We choose to count the initial onset of visible spreading, seen as the transition between the near-spherical cell initially planted (physically attached) on the substrate and the cell with adhesion processes engaged and its shape developing an inflection zone around the rim (see Fig. S2 for a more detailed illustration and explanation and Fig. S3 for an illustration in which this criterion is reached in the “standard” cell-spreading curves showing the area increase with time). This morphological transition turns out to be easily identified as the near-spherical cell has a sharp edge with interference bands in higher magnification and also a lensing effect of focusing light, which disappears in the transition to a more flattened shape. It must be emphasized that for our cell counting to be meaningful, the cells have to be isolated on the substrate; once the cells come into contact with each other, many other adhesion and mechanosensing mechanisms engage (for example, those based on cadherins), and they spread much more readily and more significantly. That is why our initial cell density was chosen so that the initial attachment is in isolation, and our spreading criterion is applied before they spread sufficiently to come in contact (as some cells in Fig. 1 have done).

We have carried out many dozens of such spreading experiments, deliberately varying the conditions: comparing cells of different generation and age (passage number), medium with and without penicillin/streptomycin, with and without CO_2_ tent, and at slightly varying pH of the medium—all on different substrates and at different temperatures. Fig. S3 illustrates the robustness and reproducibility of these experiments, which also confirms the meaningful use of the “spreading criterion.”

**Figure 3:**
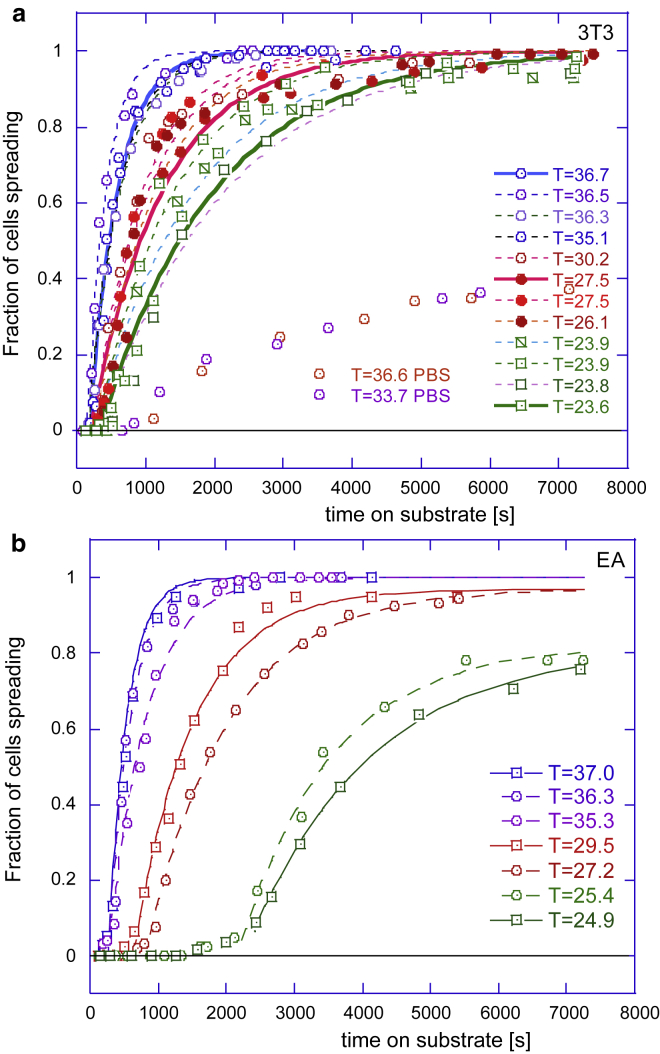
Cumulative population dynamics of cell spreading. Plots (*a*) and (*b*) show fraction of spreading cells on glass at many different temperatures for 3T3 fibroblasts and EA endothelial cells. Lines in all plots are the fits of the long-time portion of data with the exponential relaxation curves, producing the fitted values of the longest relaxation time *τ* (see text). To see this figure in color, go online.

In each individual experiment (given substrate, fixed temperature, and other parameters), once the cells were deposited on the substrate and the clock started, we took broad-field microscopic images at regular time intervals and counted the fraction of cells that have crossed the threshold defined by our spreading criterion—that is, the cells that have started the active spreading process in response to their mechanosensing cue. This produced a characteristic sigmoidal curve for each experiment (see Fig. 2); the fraction of cells engaged in spreading starting from zero at *t* = 0 and saturating at near 100% at a very long time (if we exclude the occasional cell mortality, which was more of a factor at lower temperatures). The typical sample size was 100–120 cells in each experiment (field of view); however, we have taken many similar samples and verified the high fidelity of data. The main sources of error were inconsistency of application of the spreading criterion in image analysis, imperfections of fibronectin coverage on substrate, temperature fluctuations, and, of course, the natural cell variability. All of these are random errors with no systematic drift. We were satisfied that the results were reproducible, and errors did not dominate the data trends. The plots in Figs. 2 and 3 do not include error bars not to obscure distinct data sets, but the reader could gauge this error from Fig. S3.

## Results

We first emphasize that our experiments concurred with the results of earlier studies (1, 2, 4, 26). Cells placed on stiffer substrates spread to larger areas and were less rounded for both our cell types. There is also a strong dependence on the ECM protein coverage (32), but this was not a variable in our study.

The time of initiation of spreading is presented in Fig. 2. These two plots (for 3T3 and EA cells) show the fraction of cells that have started spreading at each given time that has passed after planting on substrates and replacing the medium. The point of steepest gradient in these cumulative curves marks the most probable time for the onset of spreading (see Supporting Materials and Methods for the detailed analysis). We see the timing of cell spreading is completely insensitive to the substrate stiffness; the kinetics of a spreading response is exactly the same on each substrate. The work of Margadant et al. (33) has reported a similar effect (the rate of spreading did not depend on the degree of ECM protein coverage on the surface). Instead of substrate stiffness, we find the curves in Fig. 2 are strongly segregated by temperature.

### Long-time trend: A rate-limiting process

To examine the effect of temperature in greater detail, in Fig. 3, we plotted the same cumulative spreading fraction curves for the two cell types on glass (as we are now assured that these curves are the same on all substrates). It is noticeable that the initial lag is greater in the EA cells and that at low temperature, the saturation level drops significantly below 100%—presumably because more cells disengage (or die) at low temperatures, reducing the saturation fraction. The same effect is much enhanced for the nutrient-starved cells in the PBS medium (see in Fig. 3 *a*); the onset of spreading is very slow in this case, and a large fraction of cells do not engage at all. Nevertheless, the generic sigmoidal shape of these cumulative curves is universal, and the random spread of data within each individual experiment is not excessive. We then look to analyze the trends in this time dependence.

The curves of the generic shape seen in Figs. 2 and 3 are encountered in many areas of science, and their characteristic foot at early times, especially obvious at lower temperatures, is usually associated with a lag in the corresponding process. We will discuss this early-time regime separately, later in the article, but first, we fit exponential relaxation curves to the long-time portion of the data (as the *fit lines* in Fig. 3 indicate): *Q*(*t*) = *A* × (1 − exp[−(*t* − *t*_lag_)/*τ*]. The Supporting Materials and Methods give the table of values of *A* and *τ* for each curve, but it is clear from the plots that the fitting to the single-exponential relaxation law, with just two parameters because *A* is known for each curve, is very successful. The characteristic relaxation time *τ* markedly increases at low temperatures. It is interesting that such a characteristic time associated with the “spreading of an average cell” has been discussed in (18), giving the same order of magnitude (of the order of magnitude 50–100 s).

**Figure 4:**
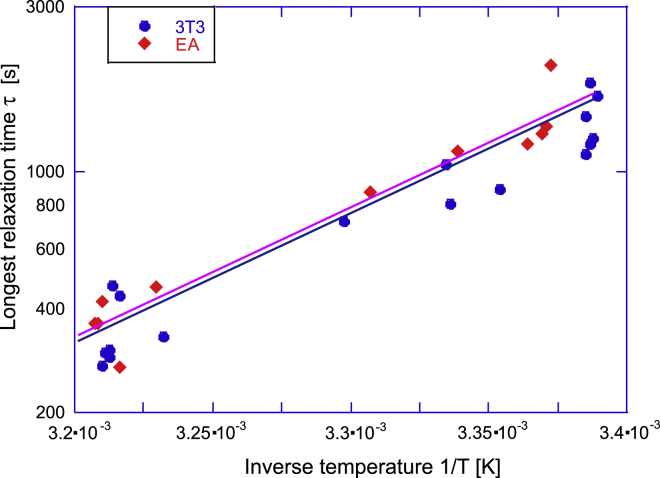
The Arrhenius plot of the longest relaxation time (log(*τ*) versus inverse absolute temperature) from the exponential fits in Fig. 3, *a* and *b*, giving almost exactly the same value of binding energy *ΔG* ≈ 18 kcal/mol for both types of cells. To see this figure in color, go online.

To better understand this dependence on temperature, we tested a hypothesis that this relaxation time is determined by the thermally activated law by producing the characteristic Arrhenius plots of relaxation times for both cell types (see Fig. 4). It is remarkable that both cells show almost exactly the same trend of their relaxation time. The rate-limiting process in their spreading pathways is the same: $\tau =\tau_{0}e^{\Delta G/k_{B}T}$, with the activation energy *ΔG* ≈ 18.3 ± 1.5 kcal/mol and the thermal rate of attempts *τ*_0_^−1^ ≈ 4 × 10^10^ s^−1^. Both values are very sensible; this magnitude of *ΔG* is typical for the noncovalent bonding energy between protein domains (34), and this rate of thermal collisions is in excellent agreement with the basic Brownian motion values.

### Early-time dynamics

**Figure 5:**
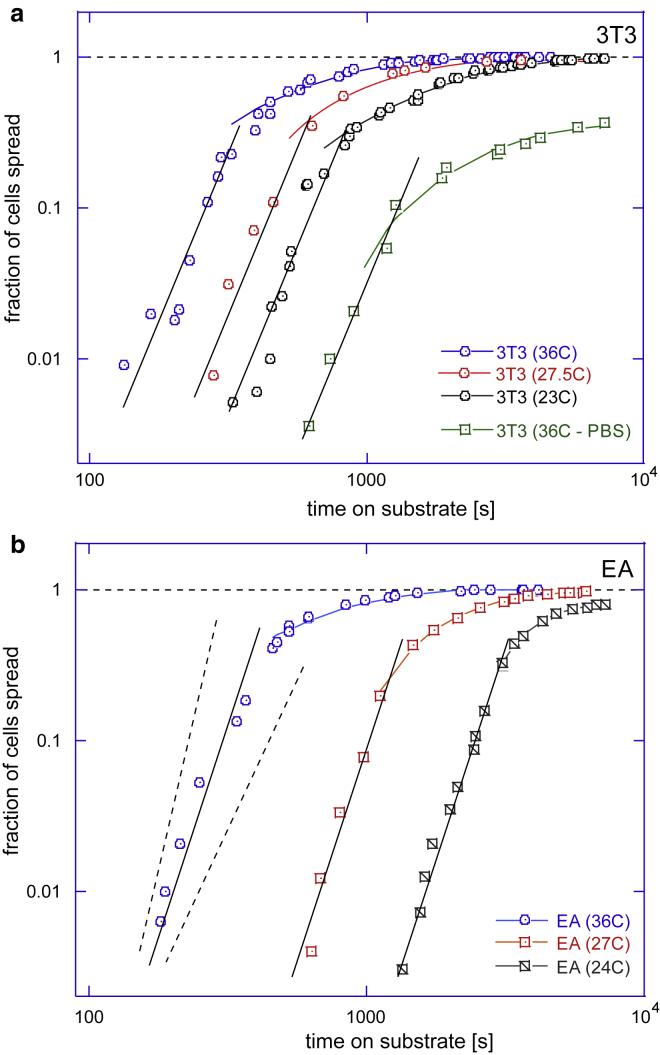
Analysis of the short-time dynamics of cell spreading. Plots (*a*) and (*b*) show selected data sets from Fig. 3, *a* and *b* presented on the log-log scale to enhance the short-time dynamical range. In both plots, the power-law slopes of the short-time data follow the equation *αt*^5^, with the coefficient prefactor *α* depending both on cell type and on temperature. The dashed line illustrates the slopes of *t*^6^ and *t*^4^ to illustrate the strength of fit. To see this figure in color, go online.

After discovering that the late-time (rate-limiting) dynamics of the onset of spreading is quite universal across different cells and substrates, it becomes clear that the marked difference between the two cell lines in Fig. 3 lies in the early-time behavior, something that we have called a “lag” after many similar situations in protein self-assembly. To examine this early-time regime more carefully, we replotted the same time series data on the log-log scale in Fig. 5.

This reveals that the process is active from the very beginning (*t* = 0), and the plotted value grows as a power law of time. The only reason that we appear to see a “lag” is because our experimental technique of counting the cells engaging in spreading did not permit values below 0.01 (1%) to be resolved in this plot; the same certainly applies to other experimental situations reporting similar kinetic data. The trend illustrated in Fig. 5 is clear; the early onset of cell spreading follows a universal power law, and the fitting of all our data sets gives *Q*(*t*) = *αt*^5^ with very good accuracy, where only the prefactor *α* depends on temperature and the cell type. We find this result truly remarkable: similar to the universal value of binding energy that controls thermally activated rate-limiting relaxation time *τ*, this very specific *t*^5^ power law appears to be the only sensible fit of the early-time data for different cells, temperatures, and substrates.

**Figure 6:**
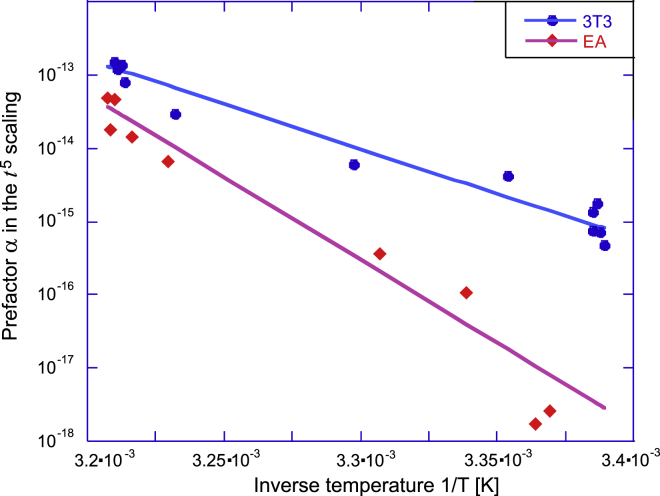
Analysis of the short-time dynamics of cell spreading. The Arrhenius plot of the prefactor *α*(*T*), with the fit lines giving the effective activation enthalpy *ΔH* ≈ 70 kcal/mol for 3T3 and 129 kcal/mol for EA. See text, which explains how this value represents the sum of free energy barriers of key proteins assembling into the adhesion complex. To see this figure in color, go online.

Again, strong temperature dependence is evident in the subpopulations of cells that start spreading very early; the difference was evident in Figs. 2 and 3 but is very clearly enhanced in Fig. 5. What changes between the data sets is the prefactor *α* of the universal power law *αt*^5^, which has a systematic temperature dependence (the fitted values of *α*(*T*) are listed in Table S2). Now expecting the thermally activated behavior, by analogy with the earlier analysis, we plot these prefactors *α*(*T*) on the Arrhenius plot in Fig. 6. The fitting to *α* = const × $e^{-\Delta H/k_{B}T}$ indeed gives a very reasonable trend with the activation energies *ΔH* = 70 kcal/mol for 3T3 and 129 kcal/mol for EA. Note that, in contrast to Fig. 4, here, we have a negative exponent (i.e., the parameter *α*(*T*)), which represents a reaction rate rather than a relaxation time. In the classical Arrhenius-Kramers thermal activation, the process time is shorter as the temperature increases, whereas Fig. 6 shows the scaling factor *α*(*T*) is decreasing as the temperature decreases instead (which is reflected in the overall observation of longer lag time in the cumulative curves).

## Discussion

The time we measure is the sum of the adhesion lag time and the time to reach our binary criterion for the start of spreading. The distribution in our data is due to the statistical distribution of the “lag times.” Our results show that the stochasticity of lag time has structure. We can use this structure to infer information about the processes underlying adhesion and spreading.

In classical physics, early-time power-law kinetics in a cumulative distribution are a hallmark of self-assembly processes, such as polymerization or aggregation (35, 36). The reason for this is that at short times, the kinetics are dictated by the number of states you must pass through to reach a final state. In this case, we must be looking at a process of self-assembly within the cell. The exponent of the power law gives us some idea of how many important assembly steps there are. But, what exactly are we assembling? To us, it seems likely that we are observing the formation of adhesion points and adhesome complexes that allow the cell to bind onto its ECM environment and begin spreading. The idea is that the initial assembly of adhesome complexes is responsible for the initial changes in the cell footprint area (e.g., Reinhart-King et al. (20)). Here, we are able to infer some quantitative details of this process.

It is well established that disruption of the integrin-fibronectin linkage completely halts cell spreading (17, 37). Integrins are transmembrane receptors linking the cell to the matrix in focal adhesions (38, 39, 40). To attach to their ligands, they need to be activated (41, 42); in isolation, integrin pairs will lie in their inactive state, unable to bind to fibronectin (or other ECM proteins containing the RGD motif). In equilibrium, the level of integrin activation might be dependent on the ECM rigidity (43, 44); however, here, we are examining very early stages of cell settling on its substrate, so it is the adhesome assembly and signaling that control our results.

Much of the literature on focal adhesions sees the attachment of the talin head domain to integrin tails as an important activation step (45, 46, 47). Talin is a key protein in mature and nascent adhesions, linking integrins to the actin cytoskeleton and providing a scaffold for other focal adhesion proteins (see, for example, (48)). For the onset of spreading, there is some conflict in the literature; in the study by Zhang et al. (17), in which they confirmed that integrin linkage was essential to the onset of spreading, they actually depleted both types of talin and found that the onset of spreading was not fully inhibited, although spreading was severely limited. This could indicate that talin was not needed for the activation of integrins during the onset of spreading. However, a subsequent knockout study of talin (among other proteins) (49) found that spreading was actually completely inhibited by the removal of talin (although partial function was restored by the addition of Mn^2+^). In that work, the authors note that the experimental methods (small interfering RNA transfection) employed in previous studies left residual amounts of proteins in the cell and that there may well have been enough talin left in depleted cells to form nascent adhesions. Indeed, in their article, Zhang et al. say that the decrease in talin2 levels (talin1 was not expressed in their cell lines) was between 40 and 68%.

In fact, Theodosiou et al. (49) implicate three further players: kindlins, paxillin, and focal adhesion kinase (FAK). This is not a new finding or point of view; since the early discovery of the key role of FAK in the integrin adhesome (40, 50, 51), it was understood that is is the FAK activation that produces the chemical cue for the subsequent cell mechanosensing pathways via Src, Rho, Rac, and Cdc42 as well as Erk (37, 52, 53, 54). Theodosiou et al. found that chemical inhibition of FAK reduced lamellopodia formation in cells to the level of kindlin knockout cells (49). The formation of these lamellopodia and the initiation of isotropic cell spreading was therefore found to be dependent on FAK activation. A recent model of FAK as a mechanosensor (55) shows how the rate of its activation is sensitive to the stiffness of substrate and the cytoskeletal pulling force. Importantly, when the force is low (as we would expect at early times before the mechanosensing pathways are activated and the cytoskeletal forces increase), this rate is controlled only by the bonding energy between its FERM and kinase domains, not the stiffness.

FAK clearly sits at the center of the adhesion signaling network (56). But the minimal composition of the whole adhesion-mechanosensing complex in the nascent adhesions as well as the rate of its assembly and turnover remain a question of active research and debate. Kindlins are known to be a necessary partner for talin in integrin activation (41, 47, 48). The F3 subdomain of a FERM domain mediates an interaction with *β*-integrin tails and “cooperates” with the talin head domain in integrin activation (57). Paxillin is another player in the adhesion network (48, 49, 56). In particular, in the nascent adhesions formed at the onset of spreading, kindlin was directly binding paxillin; paxillin was then recruiting FAK to these nascent adhesions. On the other hand, the important role of vinculin in several processes in the integrin-talin-FAK adhesion complex appears to be relevant mostly at the mature focal adhesion stage (33, 58, 59), and we believe its role is to bind different adhesion complexes into a dense focal adhesion raft.

How does this information tie in with our results? A recent molecular dynamics simulation (34) has explicitly calculated the bonding energy between FERM and kinase domains of FAK as *ΔG* ≈ 17 kcal/mol. Breaking this bond is the essential step of FAK activation. If we associate this barrier with the longest relaxation time examined in Fig. 4, the agreement of the *ΔG* values is remarkably close. According to the reaction rate theory, this energy barrier is the largest one of the assembly process because it produces the long-time “bottleneck” in the population dynamics of the onset of spreading.

**Figure 7:**
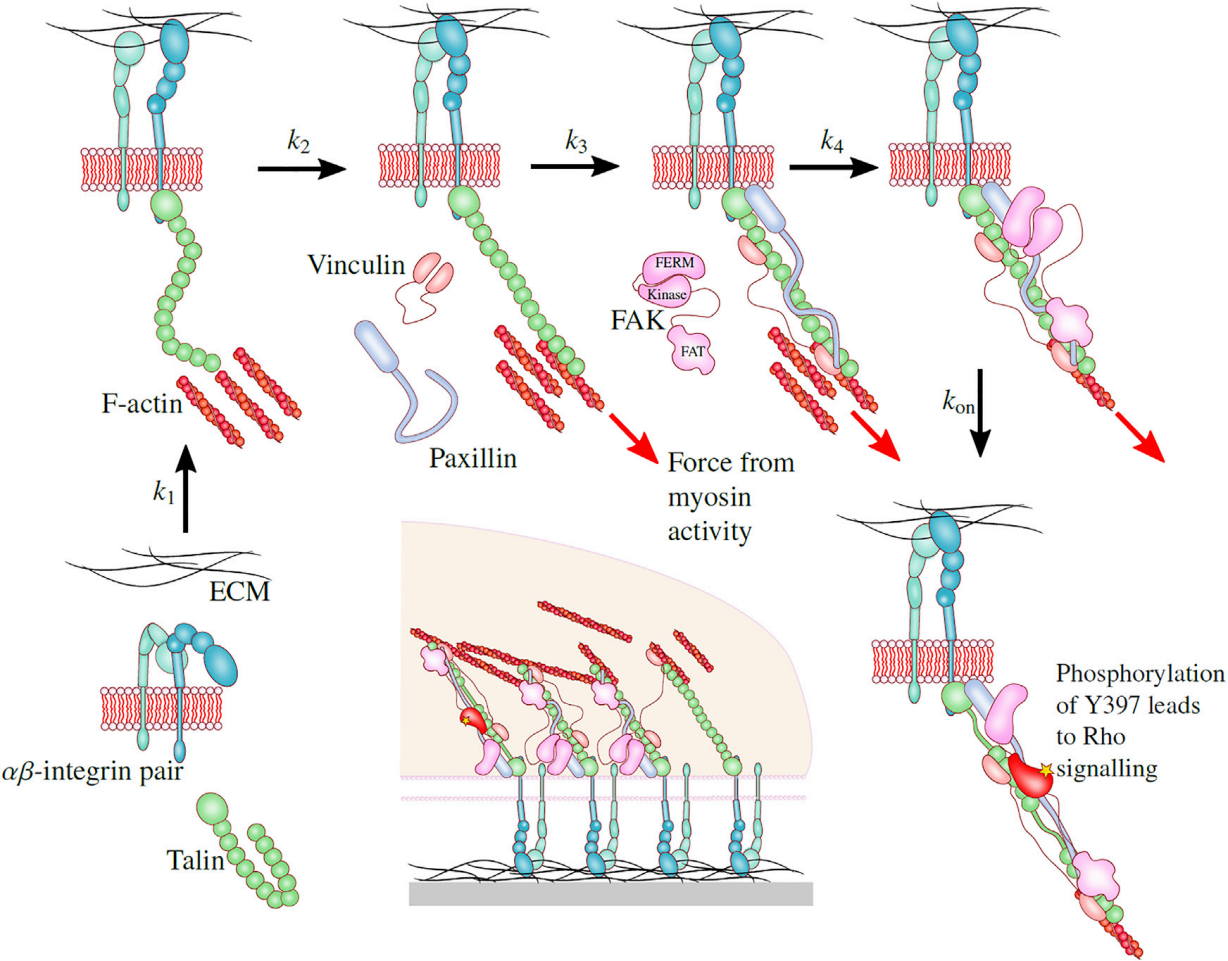
A possible assembly sequence of a mechanosensor complex. Our analysis suggests that there are five distinct slow stages illustrated in the sequence, with their respective rates *k*_1_ − *k*_4_ and the rate of FAK activation *k*_on_ (controlled by the free energy barrier *ΔG* ≈ 18 kcal/mol, cf. Fig. 4). The product of the five rate constants *α* = *k*_1_, *k*_2_, *k*_3_, *k*_4_, and *k*_on_ is what we measure in the Arrhenius plot in Fig. 6. In the center is a sketch of forming focal adhesion cluster, in which the individual mechanosensor complexes in various stages of development/turnover are bound by vinculin and actin cross-linking (see text for details and references). To see this figure in color, go online.

In a scenario in which the spreading response is initiated by the assembly of adhesome complex and the engagement of mechanosensors, the cell must undergo five changes of state before it can start spreading, with the last being the FAK activation process (55) (see Supporting Materials and Methods for detail). This is necessary for the mechanosensing signal to be generated and the cell morphological response initiated (48); it also has to be the rate-limiting step, logistically. The possible candidates for the other four reaction steps must have a rate slow enough to be counted in the first data points (see Fig. 7 for an illustration). Images of cells were taken approximately every minute, and so it is impossible to resolve fast processes with rates of *k* > 1 min^−1^ using our data. For instance, the binding of integrins to fibronectin does not fit this criterion. It has been seen that the binding of integrins to an antibody ligand in the presence of different cations has a characteristic binding time of 0.01–1 ms (60); this is much faster than we could resolve in our experimental data. To form the force-bearing chain from integrin to F-actin of cytoskeleton, we see the following reactions necessary: 1) the binding of talin and kindlin to integrins, 2) the binding of paxillin to kindlin, 3) the binding of talin to F-actin, 4) the binding of FERM domain of FAK to talin, 5) the binding of FAT domain of FAK to paxillin, and 6) the binding of FAK/paxillin to the F-actin. It is difficult to find any estimates of the rates of these processes. One can find evidence for the fast strengthening of focal adhesions under load (61), but this is not the same as the assembly of these complexes at the onset of spreading. Our experiments suggest that four of these reactions are quite slow (accounting for the need of protein localization on the complex); we cannot be certain which, but we have measured the combined activation energy of these four reactions (Fig. 6) in 3T3 and EA cells. Only once the full force chain of the integrin adhesome is assembled can the mechanosensor produce the signal for the cell to modify its morphology to the substrate.

Another possible scenario that could account for our five-step initial kinetics still has to rely on activation and adhesion of integrins but could include a phase of initial viscoelastic spreading (18) that should be controlled by physical interactions on a more macroscopic scale. In that case, we would require a few slow steps of adhesome assembly. We cannot rule this possibility out with our data, but it is interesting to note that the universal timescale suggested by Cuvelier et al. (obtained with a much lower ligand density; fibronectin coating 10 times less dense as ours) was between 5 and 10 min. Using their model with parameters they fitted for HeLa cells with our fibronectin density, gives the estimate of a spreading time to our criterion of around 2–3 min. As such, this is not inconsistent with our data, with the caveat that we are still seeing the adhesion process before spreading in the early power-law kinetics. It is also unclear whether there should be an Arrhenius activation-type temperature dependence for their spreading timescale (which is prominent in our data). Certainly, the work of Cuvelier et al. avoids kinetic complications by simply considering the adhesion energy gain per unit area of the cell.

The unusual feature of this work is the use of population dynamics of spreading cells to infer details of the microscopic processes governing the cell response to an external substrate. By linking the results to nucleation theory, details of which are given in Supporting Materials and Methods, we found a, to our knowledge, novel way of looking at the onset of cell spreading as a problem of complex assembly.

## Author Contribution

A.-L.R. carried out all experiments. S.B., A.-L.R., and E.M.T. carried out different elements of data analysis. S.B. and E.M.T. wrote the article.

## Supporting Material

Document S1. Supporting Materials and Methods, Figs. S1–S6, and Tables S1–S2

Document S2. Article plus Supporting Material
