# Supplementary material for: Universal Kinetics of the Onset of Cell Spreading on Substrates of Different Stiffness
Source: Biophys J. 2019 Jan 5;116(3):551–9. doi: 10.1016/j.bpj.2018.12.020 (PMC6369430; doi:10.1016/j.bpj.2018.12.020)
Supplement: Document S1. Supporting Materials and Methods, Figs. S1–S6, and Tables S1–S2 [file mmc1.pdf]

Biophysical Journal, Volume *116*

Supplemental Information

Universal Kinetics of the Onset of Cell Spreading on Substrates of Different Stiffness

Samuel Bell, Anna-Lena Redmann, and Eugene M. Terentjev

Universal kinetics of the onset of cell spreading: Supplementary Information

Samuel Bell, Anna-Lena Redmann and Eugene M. Terentjev[1]
Cavendish Laboratory, University of Cambridge, Cambridge, CB3 0HE, U.K.

Cells and cell culture procedures

There are different types of endothelial and fibroblast cells available. Primary cells are directly taken from donor tissue and then grown in cell culture conditions. They can be grown in culture for a specific amount of time before they undergo senescence and die. The advantage of primary cells is that they are as close to in-vivo cells as possible, but as they are taken from different donors, their behaviour is less reproducible. Immortalized cell lines are obtained from primary cells by, for example, transfection or fusion. This results in a change in their DNA, leading to indefinite proliferation. This makes it easier to handle them in multiple long-term experiments, and makes such experiments more reproducible, but at the same time many immortalized cells have some tumorous behavior[1,2]. We used immortalized cell lines: NIH/3T3 murine fibroblasts (obtained from ATCC) and EA.hy927 endothelial cells.

Cells were normally cultured at 37C and 5% CO2 in Dulbecco's modified Eagle's medium (DMEM), from Greiner, with 10% fetal bovine serum and 1% Pen/Strep (solution stabilized, with 10,000 units penicillin and 10 mg streptomycin/mL), from Sigma Aldrich. For a comparative study of the role of nutrient in the medium during the spreading experiments, we also used phosphate-buffered saline (PBS), from Thermo Fisher Scientific. Cells were subcultured in DMEM every 3 days, at about 70% confluency, by trypsinization, to avoid the formation of big lumps of cells, thus ensuring that we maintain a single cell suspension. Cells were trypsinized for 5 min (Trypsin-EDTA 0.05%). The solution was then neutralized by added complete growth medium and centrifuged at 1000 rpm for 5 min. We tested our results on several parallel cell cultures that did not use Pen/Strep, and confirmed no significant difference in our results.

The use of Pen-Strep can be questioned. Antibiotics have been used prophylactically to prevent bacterial infections in cell culture for many years, and they are still being used. It was the introduction of antibiotics that allowed the widespread development of cell culture methods in the first place, as bacterial contamination was a major problem[3]. However, although toxicity experiments found that antibiotics were harmless to mammalian cells[4], there are concerns about the use of antibiotics in cell culture associated with a neglect of aseptic technique and possible side effects of antibiotics. Many adhesion strength studies use Pen/Strep or other antimycotic or antibiotic solutions in the cell culture, and we followed this procedure as well. We have tested our results on several parallel cell cultures that did not use Pen/Strep, and confirmed that no significant difference was inflicted on our results.

Substrates of varying stiffness

The plot in Fig. S1 shows the data from a standard frequency-sweep rheology test, in an oscillating parallel-plate rheometer (Anton Paar) at constant temperature of 25°C and low shear strain of 3% (after separately testing that this level of strain remains well within the linear-elastic regime). The frequency sweep shows the low-frequency rubber plateau of the storage modulus $G'(\omega)$, and an onset of the viscoelastic rise for the weaker gels. The usual dissipation peak, corresponding to the Rouse frequency of the average network strand, is expected to occur at 10-100 kHz. Importantly for us, the limit of equilibrium shear modulus is unambiguously reached in such a test. The values of the corresponding Young modulus are labelled in brackets for each curve (it is strictly three times the measured shear modulus because these elastomers are incompressible).

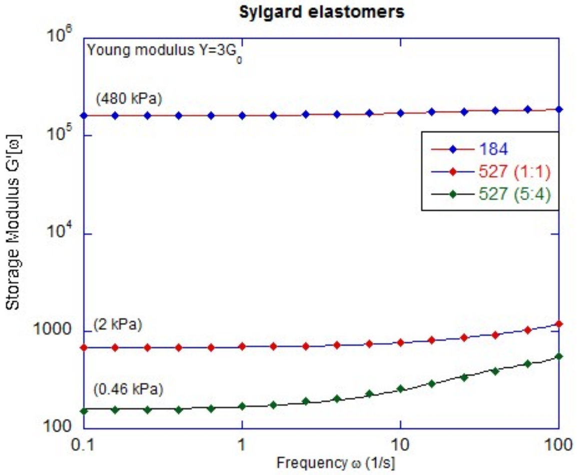

FIG. S1. The results of the rheometry testing of Sylard elastomer substrates: the storage shear modulus $G'(\omega)$ measured in the parallel-plate geometry at constant temperature of 30C. In all cases we are definitely on the low-frequency rubber-modulus plateau. Three materials are displayed: the standard Sylgard 184 elastomer, and two versions of Sylgard 527 elastomers, with 1:1 and 5:4 w/w ratio of the compound to hardener. The value of the equilibrium modulus is labelled on each curve.

It is interesting to note that three orders of magnitude in the magntitude of Young modulus are covered between the different Sylgard elastomer samples in Fig. S1, while the modulus of a standard laboratory glass slide is known from separate measurements to be of the order of 30 GPa, i.e. another 4 orders of magnitude higher than in Sylgard 184.

The spreading criterion

Establishing the unambiguous criterion, by which the cells can be labelled as "spreading" or not, is an important step in our analysis. As in several important publications on the individual dynamics of cell spreading[5–8], we are looking at the point in time, for each individual cell, when its shape crosses over the shape with a contact angle $\sim 90°$. Practically, at this point the bright-field microscope image stops having the 'lensing effect' of focusing the illuminating light in the center of the cell image, which for us was the most distinct criterion in the population analysis, Fig. S2.

The interested reader should also check the paper by Frisch and Thoimine[9], where their study of the dynamics of individual cell spreading passes through this precise stage: when the contact angle changes from a large value (above 90°, indicating partial dewetting) to a small value (below 90°, indicating partial wetting). They also quote this transition as the region where the cell cortical tension is approximately mathced by the cell adhesion energy (both of the order $2 - 4 \cdot 10^4 \mathrm{J/m}^2$).

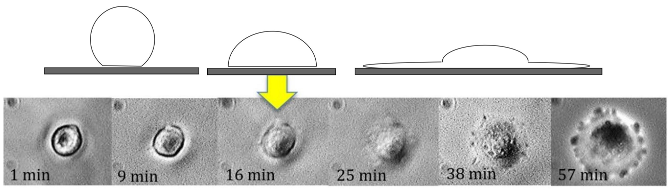

FIG. S2. The illustration of the visual criterion used to count the cells as 'engaged' in mechanosensing response. We interpreted the loss of the sharp edge in the microscopic image, and the simultaneous loss of the 'lensing effect' with the cell shape becoming flatter than semi-sphere. This point in time was associated with the given cell beginning to spread, and therefore counted towards the 'fraction of cells engaged in spreading' in the population.

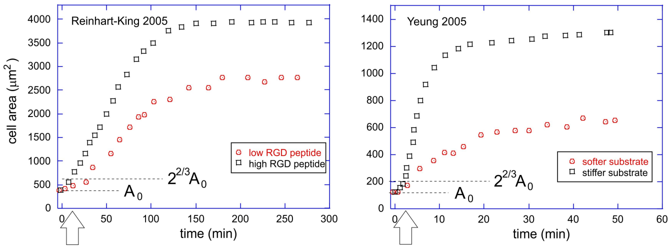

FIG. S3. Plotting some digitized data from Reinhart-King 2005 (fig.2a), and from Yeung 2005 (fig.5d) showing the time evolution of spreading cell area: in the first case at varying concentration of ligands for integrin binding, in the second case at varying substrate stiffness. The initial cell area (on first deposition on surface) is A_0, and our 'selection criterion' illustrated in Fig. S2 implies the observed cell area increases by a factor $2^{2/3} \approx 1.6$. This is a very early stage of cell spreading, which we refer to as the onset of spreading response.

In order to maximize the sampling size, in our main experiments we have chosen a low magnification and broad field of view, to include around 100 individual cells on the substrate, without contact with each other. At low magnification, the lensing effect is quite pronounced, and so we were able to count the number of cells engaged in spreading in each time-lapsed image without moving the sample.

Quality and reproducibility of data

Our raw data comes in the form of a 'spreading fraction', which counts the number of cells in each experiment (with a total in the range of ca. 100 cells in a constant field of view each time), which have passed the spreading criterion at each given time. In order to be able to subject this data to rigorous quantitative analysis, we must be sure it is reproducible, and have a good assessment of uncertainty in each value of 'spreading fraction'. To this end, we have performed many dozens of separate experiments to assure the reproducibility of their results. For each individual spreading curve (for a given temperature, cell type, substrate, and other conditions) the error bars were quite small – we decided not to include them in the key plots in the main text (Figs. 2 and 3) because they would obscure the distiction between different curves, which was the main point in those plotes.

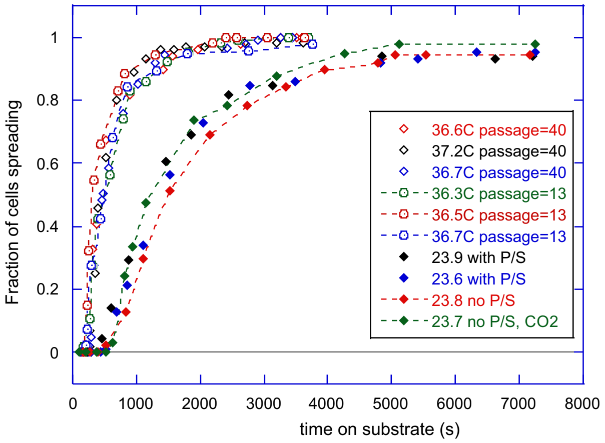

FIG. S4. Comparing the results of a spreading experiment for 3T3 cells of very different passage number, with and without Penn-Strep, and without CO2 tent (all constituting small variations of experimental conditions). The temperature labelled in the plot was the constant actual temperature during the experiment. The error bars, statistically calculated for each data set would be about a half of the spread in the plot.

Here we give an illustration of the robustness of our quantitative data by showing several comparisons. Figure S4 shows the spreading curves obtained in ten separate experiments on 3T3 cells on glass/fibronectin. There are two groups of data (and curves) at two temperatures, aiming to be 37C and 24C. Our device was able to main-

tain the constant temperature during the whole experiment duration due to an active feedback heater system – however, the precision in setting the temperature in advance of each experiment was not greater then $\pm 1°$, hence a spread in actual T-values. We did not regard this variation exceeding the other experimental uncertainties, and in the main text only referred to temperatures with two significant figures.

At the high temperature of 37C, the difference between data sets was in the 'age' of cells: the {young} kind refers to 3T3 cells with the passage number 13, compared to the {old} cells with the passage number 40. Clearly, within this typical range of practical usefullness of reproducing fibroblast cells there data is reproducible. At the lower temperature of 24C we show experiments where we changed the medium environment: with and without Pen/Strep antibiotic, and with a slightly different pH due to removing the standard CO2 tent. In all cases, in spite of such a variation of experimental parameters, the spreading curves are clearly reproducible, and their random variation is not high. The conclusions were the same for EA cells. Therefore, we are assured that the cell counting using our 'spreading criterion' was robust, and fitting of these curves to the model equations reliable.

Fitting data for the long- and short-time relaxation

At long times in our population dynamics we have fitted the single-exponential law of relaxation to saturation, in which all cells are declared 'spreading'. In each case using a fixed offset of the time origin by the 'lag time' (see Fig.3 of the main text), using the equation $f(t) = A \cdot (1 - \exp[-(t - t_{\rm lag})/\tau])$ in two stages: first setting the amplitufe A manually from the apparent saturation level, and using the two-parameter fit to obtain $t_{\rm lag}$ and τ, then fixing $t_{\rm lag}$ and using the two-parameter fit to obtain the saturation amplitude A and the relaxation time τ, which are listed in the table S1. It has turned out that this two-step fitting produced the values of A not differing by more than 5% from the ones we initially set manually, which reassures us in the high quality of this fitting, and the resulting Arrhenius analysis of thermally-activated rate-limiting relaxation presented in Fig. 4 of the main text.

In contrast, in the detailed analysis of initial stages of the cumulative spreading curves in our population dynamics, when only a few cells are beginning to spread (the short-time onset of spreading fraction, see Fig. 5 of the main text), we have discovered that there is no such thing as a 'lag' in this process. The time-resolution of our experiment was naturally limited at very short times: we could not start taking images sooner than 2 min after their planting (since the original culture had to be replaced with the clean medium). Nevertheless, the logarithmic time-axis in Fig.5 of the main text allows a clear view of the power-law regime. We found that the fraction of cells engaged in their spreading process was fitted to the power-law with the fixed power t^5. This has

T(C)	3T3: τ[s]	Max: A	T(C)	EA: τ[s]	Max: A
36.8	275 ± 20	0.99	37	310 ± 21	1.00
36.7	298 ± 19	0.99	36.9	363 ± 27	0.99
36.6	290 ± 20	0.99	36.8	421 ± 23	1.00
36.6	306 ± 18	1.00	36.3	271 ± 21	0.99
36.5	465 ± 22	0.98	35.3	461 ± 33	0.97
36.3	435 ± 35	0.96	29.5	873 ± 58	0.96
35.1	333 ± 28	1.00	27.2	1141 ± 23	0.96
30.2	716 ± 59	0.98	25.4	1201 ± 357	0.81
27.5	1051 ± 82	0.95	25	1282 ± 212	0.81
27.4	804 ± 77	0.99	24.9	1348 ± 228	0.79
26.1	891 ± 82	0.98	24.8	2023 ± 329	0.89
23.9	1122 ± 98	1.00			
23.9	1452 ± 110	0.96			
23.8	1205 ± 103	0.94			
23.8	1815 ± 128	0.98			
23.7	1252 ± 112	0.95			
23.6	1655 ± 143	0.92			

TABLE S1. Values of the parameters of the fitted equation $Q(t) = A(1 - \exp[-t/\tau])$, for different temperatures, and two studied cell types. These values of $\tau(T)$ were used to produce the Arrhenius plot in Fig. 4 in the main text.

T(C)	3T3: α	T(C)	EA: α
36.8	1.51e-13	37	4.93e-14
36.7	1.19e-13	36.9	1.79e-14
36.6	1.34e-13	36.8	4.56e-14
36.6	1.14e-13	36.3	1.44e-14
36.5	8.04e-14	35.3	6.65e-15
36.3	1.03e-13	29.5	3.65e-16
35.1	3.00e-14	27.2	1.07e-16
30.2	5.90e-15	25.4	1.75e-18
27.5	3.73e-15	25	2.63e-18
27.4	3.32e-15	24.9	8.05e-18
26.1	4.28e-15	24.8	5.50e-18
23.9	9.10e-16		
23.9	1.36e-15		
23.8	7.59e-16		
23.8	1.74e-15		
23.7	7.20e-16		
23.6	4.75e-16		

TABLE S2. Values of the prefactor in the universal power-law fitting of short-time data: $Q(t) = \alpha\, t^5$ (for the time taken in seconds), for different temperatures, and two studied cell types. These values of $\alpha(T)$ were used to produce the Arrhenius plot in Fig. 6 in the main text.

been done in two stages: we first fitted the data to an arbitrary power-law αt^{β}, with two fitting parameters α and β, establishing that the exponent β is always close to five. Then we enforced the strict $\beta = 5$ condition, and obtained a fit with just one free parameter α, changing with temperature, which is listed in the table S2.

FAK as reversible mechanosensor

To probe the mechanical modulus of a medium, a force has to be applied to it, either as a local point source, or as distributed stress. The source of this force is the actin-myosin activity of the cytoskeleton delivered via the ATP-rich barbed terminus of F-actin. We need to trace the series of connected devices, from the point of force origin (F-actin) to the point of its application to the ECM. Figure 7 in the main text illustrates this force chain along the assembled protein complex, which has been reproduced in a large number of important publications in this field[10,11].

The integrin family of transmembrane proteins link the extracellular matrix (ECM) to the intracellular actin cytoskeleton via a variety of protein-tyrosine kinases, one of which is the focal adhesion kinase (FAK). Integrins are aggregated in focal adhesions, but at the early stages of activation of cell adhesion mechanisms the focal adhesions are not yet formed. Activation of integrins is required for binding to the ECM proteins: active integrins acquire ligand affinity. It is well established that integrin activation and clustering leads to FAK activation and the subsequent signaling chain of mechanosensing and cytoskeletal remodeling, e.g. see the review by Parsons[12]. There is a large body of literature on integrins, with definitive reviews by Hynes[13,14] explicitly stating that integrins are the mechanosensors. It has recently been demonstrated that the integrin bond with fibronectin has catch-bond characteristics[15], and therefore could have a graded response to force and stiffness.

However, activated integrins possess no catalytic activity, and so cannot act as a mechanosensor on their own. A good summary by Giancotti[16], while talking about integrin signalling, in fact shows schemes where FAK is the nearest to cytoskeletal actin filaments. The important work by Guan et al.[17] establishe a clear correlation chain of extracellular fibronectin transmembrane integrins intracellular FAK, but offers no reason to assume that integrin is the sensing device on this chain. There is a clear indication that phosphorylation of FAK is a key step in the mechanosensing process, e.g. see the review by Geiger et al.[18], pointing at FAK as a fulcrum of this tapestry. Indeed, Schaller et al.[19] state that FAK phosphorylation is the initial step of signaling, and show evidence that crosslinking integrins and ECM (i.e. making the 'substrate' stiffer) leads to an enhanced FAK phosphorylation, while conversely, a damage to integrin is connected with a reduced activation of FAK.

The application of cytoskeletal tension in mechanosensing at focal adhesions is now well established[11]. A key role in this system is played by talin. There are many papers investigating the correlation of talin (as well as paxillin) with β-integrin and FAK; recent studies clearly show that talin is capable of high stretching by a tensile force[20], implying a function similar to that of titin in muscle cells: acting as an extension-limiter. It is also now clear that the immobile domain at the N-terminal of talin is associated with integrin, and also closely associated with the FERM domain of FAK[15], while the C-terminal of talin is associated with paxillin, which in turn may associate with the focal adhesion targeting (FAT) domain (C-terminal) of FAK. Both talin and paxillin also bind to cytoskeletal F-actin. These actin filaments exert a pulling force on the C-terminal of talin, making it play a role of a scaffold for other proteins to arrange around. More importantly, this allows the pulling force to be transmitted from the cytoskeleton to the ECM. This could be used to effect the conformational change in FAK required for its activation. In this model, integrin is merely the bridging element from FAK to the ECM, with the FERM domain localized near the cell membrane and N-terminal of talin. At the opposite end, the FAT domain can be pulled away by the cytoskeletal force transmitted through paxillin/talin. This model is supported by the recent computational study[21], showing that the closed and the open states of FAK are reversibly reached by increasing and decreasing of pulling force.

In our recent theoretical work[22], using this idea of FAK conformational change under applied force, we demonstrated that sensing of stiffness may be a distinct single-molecule response, and develop a theoretical model of reversible mechanosensor. The underlying physics of our model is applicable to a wide variety of protein complexes, but we concentrate on FAK as it occupies a central point in mechanosensing pathways of focal adhesions. The activation of FAK is dependent on cytoskeletal tension, and on ECM stiffness, while the integrin (along with other members of the force chain) is merely playing a role of force transducer. Of course, without the activated integrin there would be no force transduction to ECM, and no mechanosensing. We do not consider the role of clustering into focal adhesions for the early stages of adhesion mechanisms activation.

Nucleation theory leading to power law scaling

At short times, all of our curves exhibit a characteristic power-law dependence on time remarkably, with the same exponent for both types of cells and at all temperatures. In the literature on various kinetic processes, this early-time region is often mistakenly called the 'lag time'; it is clear that there is no such thing as lag just a steep power-law with a possibly high exponent achieves this apparent delay of the growth phase.

Historically, the first time such a process was ana-

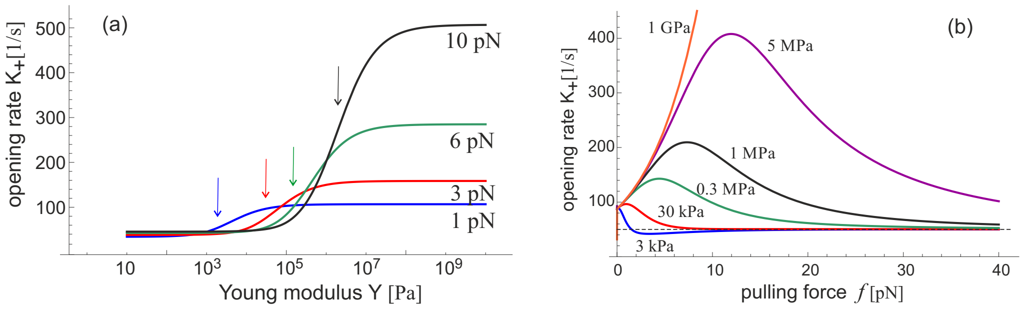

FIG. S5. (a) The rate constant of the FAK activation transition $K_+(f, \kappa)$ plotted against the substrate stiffness (on logarithmic scale) for several values of pulling force f. The arrows point at the inflection point on each curve, i.e. the region of maximum sensitivity. (b) The rate constant $K_+(f, \kappa)$ is plotted as a function of the cytoskeletal pulling force f, for several values of substrate stiffness labelled on the plot. The homeostatic peaks of activation rate $K_+(f)$, for each given substrate stiffness, roughly correspond to the peak of sensitivity in plot (a) at the same level of force. This suggests that the cell self-adjusts the sensor to keep it at the optimal sensitivity on each substrate.

lyzed, and the power-law theoretically reproduced, was in the context of protein self-assembly (gelation of deoxyhemoglobin) by Eaton et al.[23]. They have considered the critical nucleation process, when the aggregating monomers need to reach a nucleus size n_c by adding new particles against an energy barrier while when the size of an aggregate exceeds n_c, the fast polymerization ensues. By assuming the growth of the critical nucleus via a single monomer addition with the same forward rate k_f, it is easy to show by iterative integration that the concentration of the aggregate of N monomers grows at short times as:

$$c_N \approx c_1 \frac{(k_f t)^{n_c - 1}}{(n_c - 1)!}, \tag{1}$$

where c_1 is the initial concentration of monomers. At longer times, additional terms become important, and the rate of growth slows down and saturates when the process is concentration-limited. This analysis is, in turn, similar to the one used by Abraham in the study of kinetics for the nonsteady-state nucleation of supersaturated water vapor. The simple conclusion is: if the kinetics requires an initial assembly of a nucleus made of n_c particles the early time dependence will be the power law with the exponent $(n_c - 1)$, one power per each monomer addition reaction, and the prefactor proportional to the product of rates of individual monomer addition to the growing nucleus.

In fact, the above example of nucleation rate is a particular case of a more general problem in the network theory. Let there be a set of discrete states $\{S_i\}$. These can be represented as nodes of the network, and the transition processes that link these states can be represented as directed edges between these nodes. The process taking the cell from state S_i to state S_j proceeds with a rate k_{ij}. Such a network may have a complex topology, which does not affect the core result we are leading towards. Defining the transition rate matrix of the network, $(\mathbf{Q})ij = k_{ij}$, the probability for the whole process can be written as a vector, $\mathbf{P}(t)$, with each component corresponding to a state Si at time t. Using the Markov chain theory, one can write down the time-dependence of $\mathbf{P}(t)$:

$$\frac{d\mathbf{P}}{dt} = \mathbf{Q} \cdot \mathbf{P} \tag{2}$$

This is called the backwards Kolmogorov equation. If we let state S_0 be the initial state of the process, then the initial condition, on the probability $\mathbf{P}$ at time $t = 0$, is that the first element, corresponding to the probability of being in S_0, is equal to one, and all other elements are zero.

It turns out that at short times, the probability of reaching the final state S_f depends on the length of the shortest path of transitions between S_0 and S_f. A path is defined as the sequence of states the cell passes through as it evolves in time. The probability that the cell will reach the final state, $f(t)$, is given by, at short times:

$$f(t) \approx \frac{k_{01} k_{12} k_{23} ... k_{m,f}}{m!} t^m \tag{3}$$

where we label the intermediate states in the process: $[S_0] \to S_1, S_2, , S_m \to [S_f]$, such that the k_{ij} are the rates of the intermediate processes[24]. This is the probability of first passage time at small times, but our experimental data gives the cumulative probability of having reached the final state:

$$P(t) = \int_0^t f(t') \mathrm{d}t' \approx \frac{k_{01} k_{12} k_{23} ... k_{m,f}}{(m+1)!} t^{m+1} \tag{4}$$

for short times. Thus, the cumulative probability distribution shows power-law behavior at short times, with the exponent being the minimum number of thermally activated transition processes (the shortest transition pathway) necessary to get from the initial state to final state. At long times, conversely, the first passage time distribution is dominated by the longest timescale in the system (i.e. the system will bottleneck at the slowest transition process). Then, the cumulative probability reverts

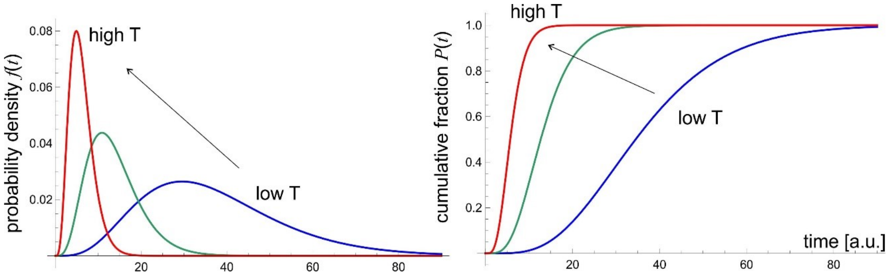

FIG. S6. The illustrations of theoretically predicted probability density $f(t)$ and cumulative fraction $Q(t)$ of mechanosensing activation, based on the crude joining together of the short- and long-time results: the $f(t) = \mathrm{const} \cdot t^4 e^{-t/\tau}$, while the cumulative fraction reproduces the observation data very well.

to the typical two-state single exponential decay as we see in Fig. 4 in the main text.

The critical nucleation kinetics is obviously a very simple example of the general theory, with the sequential addition of monomers to the growing nucleus being the only transition pathway with $m+1 = (n_c - 1)$ steps. Examining the adhesion-mechanosensing complex in Fig. 7 of the main text, discussed in much greater detail in[22], we see that in order to complete the chain of transmitted force from the cytoskeletal F-actin to the adhesion point in the ECM several proteins have to assemble into the complex: The dimer of α- and β-integrins needs to bind to talin, which is needed to activate the integrin, and also provides the anchor for the FERM domain of focal adhesion kinase; at the other end, paxillin and talin are needed to mediate the binding of FAT domain. Our kinetic analysis suggests that there are exactly $n_c = 6$ key players in this complex. Each of these thermally activated binding events has its own rate k_{ij} , and the analysis suggests that the sum of the five energy barriers is 70 kcal/mol for 3T3 fibroblasts, and 130 kcal/mol for endothelial cells. It makes sense to have the rates of adhesion-mechanosensing complex assembly higher in fibroblasts, which have a much more prominent mechanical function. It is possible that this is achieved by an additional companion protein assisting one or several of these binding events.

The main experimental data in this work consists of the cumulative curves, which represent the fraction of cells that have engaged in their mechanosensing response at a given time. We now know that the early-time section of this data is well represented by a t^5 power law, while the late times reflect the simple exponential decay towards the saturation plateau. Importantly, the local gradient of this cumulative curve has the meaning of probability density for the cell to engage at a given time, and combining the two time-regimes together we can illustrate this probability in Figure SS6 with the probability density $f(t) = \mathrm{const} \cdot t^4 e^{-t/\tau}$, and the cumulative fraction $Q(t) = \int_0^t f(t')\mathrm{d}t'$. The maximum of the probability density marks the time at which the cell mechanosensing activation is most likely to occur, while the mean activation time is given by the usual $\langle t \rangle = \int_0^\infty t f(t)\mathrm{d}t$.

[1] D. Bouïs, G. A. P. Hospers, C. Meijer, G. Molema, and N. H. Mulder, "Endothelium in vitro: a review of human vascular endothelial cell lines for blood vessel-related research," Angiogenesis **4**, 91–102 (2001).
[2] R. I. Freshney, *Culture of Animal Cells* (John Wiley & Sons, Inc., 2010).
[3] I. Kuhlmann, "The prophylactic use of antibiotics in cell culture," Cytotechnology **19**, 95–105 (1995).
[4] C. N. D. Cruickshank and E. J. L. Lowbury, "Effect of antibiotics on tissue cultures of human skin," Brit. Med. J. **2**, 1070–1072 (1952).
[5] H.-G. Döbereiner, B. Dubin-Thaler, G. Giannone, H. S. Xenias, and M. P. Sheetz, "Dynamic phase transitions in cell spreading," Phys. Rev. Lett. **93**, 108105 (2004).
[6] Y. Xiong, P. Rangamani, M.-A. Fardin, A. Lipshtat, B. Dubin-Thaler, O. Rossier, M. P. Sheetz, and I. R., "Mechanisms controlling cell size and shape during isotropic cell spreading," Biophys. J. **98**, 2136–2146 (2010).
[7] J. Li, D. Han, and Y.-P. Zhao, "Kinetic behaviour of the cells touching substrate: the interfacial stiffness guides cell spreading," Sci. Rep. **4**, 3910 (2013).
[8] D. Cuvelier, M. Théry, Y.-S. Chu, S. Dufour, J.-P. Thiéry, M. Bornens, P. Nassoy, and L. Mahadevan, "The universal dynamics of cell spreading," Curr. Biol. **17**, 694–699 (2007).
[9] T. Frisch and O. Thoumine, "Predicting the kinetics of cell spreading," J. Biomech. **35**, 1137–1141 (2002).
[10] V. P. Hytönen and B. Wehrle-Haller, "Mechanosensing in cell–matrix adhesions–converting tension into chemical signals," Exp. Cell Res. **343**, 35–41 (2016).
[11] G. Giannone and M. P. Sheetz, "Substrate rigidity and force define form through tyrosine phosphatase and kinase pathways," Trends Cell Biol. **16**, 213–223 (2006).
[12] J. T. Parsons, "Focal adhesion kinase: the first ten years," J. Cell Sci. **116**, 1409–1416 (2003).
[13] R. O. Hynes, "Integrins: versatility, modulation, and signaling in cell adhesion," Cell **69**, 11–25 (1992).
[14] R. O. Hynes, "Integrins: bidirectional, allosteric signaling machines," Cell **110**, 673–687 (2002).
[15] F. Kong, A. J. García, A. P. Mould, M. J. Humphries, and C. Zhu, "Demonstration of catch bonds between an integrin and its ligand," J. Cell Biol. **185**, 1275–1284 (2009).
[16] F. G. Giancotti, "Complexity and specificity of integrin signalling," Nat. Cell Biol. **2**, E13–E14 (2000).
[17] J.-L. Guan and D. Shalloway, "Regulation of focal adhesion-associated protein tyrosine kinase by both cellular adhesion and oncogenic transformation," Nature **358**, 690–692 (1992).
[18] B. Geiger, J. P. Spatz, and A. D. Bershadsky, "Environmental sensing through focal adhesions," Nat. Rev. Mol. Cell Biol. **10**, 21–33 (2009).
[19] M. D. Schaller, C. A. Borgman, B. S. Cobb, R. R. Vines, A. B. Reynolds, and J. T. Parsons, "pp125-fak, a structurally distinctive protein-tyrosine kinase associated with focal adhesions," Proc. Natl. Acad. Sci. USA **89**, 5192–5196 (1992).

[20] M. Yao, B. T. Goult, H. Chen, P. Cong, M. P. Sheetz, and J. Yan, "Mechanical activation of vinculin binding to talin locks talin in an unfolded conformation," Sci. Rep. **4**, 4610 (2014).
[21] J. Zhou, C. Aponte-Santamaría, S. Sturm, J. T. Bullerjahn, A. Bronowska, and F. Gräter, "Mechanism of focal adhesion kinase mechanosensing," PLoS Comp. Biol. **11**, e1004593 (2015).
[22] S. Bell and E. M. Terentjev, "Focal adhesion kinase: the reversible molecular mechanosensor," Biophys. J. **112**, 2439–2450 (2017).
[23] J. Hofrichter, P. D. Ross, and W. A. Eaton, "Kinetics and mechanism of deoxyhemoglobin s gelation: a new approach to understanding sickle cell disease," Proc. Natl. Acad. Sci. USA **71**, 4864–4868 (1974).
[24] A. Valleriani, X. Li, and A. B. Kolomeisky, "Unveiling the hidden structure of complex stochastic biochemical networks," J. Chem. Phys. **140**, 064101 (2014).
