# Supplementary material for: Universal Kinetics of the Onset of Cell Spreading on Substrates of Different Stiffness
Source: Biophys J. 2019 Jan 5;116(3):551–9. doi: 10.1016/j.bpj.2018.12.020 (PMC6369430; doi:10.1016/j.bpj.2018.12.020)
Supplement: Document S2. Article plus Supporting Material [file mmc2.pdf]

Biophysical Journal
Article

Biophysical Society

Universal Kinetics of the Onset of Cell Spreading on Substrates of Different Stiffness

Samuel Bell,[1] Anna-Lena Redmann,[1] and Eugene M. Terentjev[1,*]
[1]Cavendish Laboratory, University of Cambridge, Cambridge, United Kingdom

ABSTRACT When plated onto substrates, cell morphology and even stem-cell differentiation are influenced by the stiffness of their environment. Stiffer substrates give strongly spread (eventually polarized) cells with strong focal adhesions and stress fibers; very soft substrates give a less developed cytoskeleton and much lower cell spreading. The kinetics of this process of cell spreading is studied extensively, and important universal relationships are established on how the cell area grows with time. Here, we study the population dynamics of spreading cells, investigating the characteristic processes involved in the cell response to the substrate. We show that unlike the individual cell morphology, this population dynamics does not depend on the substrate stiffness. Instead, a strong activation temperature dependence is observed. Different cell lines on different substrates all have long-time statistics controlled by the thermal activation over a single energy barrier $\Delta G \approx 18$ kcal/mol, whereas the early-time kinetics follows a power law $\sim t^5$. This implies that the rate of spreading depends on an internal process of adhesion complex assembly and activation; the operational complex must have five component proteins, and the last process in the sequence (which we believe is the activation of focal adhesion kinase) is controlled by the binding energy ΔG.

INTRODUCTION

Matrix stiffness is known to affect cell size and morphology (1–3). When cells are plated onto soft substrates, their footprint will not increase as much as on stiff substrates, and their spreading will be more isotropic; the resulting cells will be round and dome-like in shape. On stiff substrates, the same cells will spread very strongly, develop concentrated focal adhesion clusters and stress fibers of bundled F-actin, and eventually polarize to initiate migration. This leads to several well-documented biological functions in tissues: variable stem-cell differentiation pathways (1,4), the fibroblast-myofibroblast transition near scar tissue (5–7), fibrosis in smooth-muscle cells near rigid plaque or scar tissue (8,9), and the stiffer nature of tumor cells (10,11). The definitive review (12) summarizes this topic.

The actual process of spreading, after a planktonic cell is deposited on a substrate, involves several stages. After initial anchoring, which probably occurs because of a nonspecific hydrophobic or van der Waals binding, one could see an initial increase of the cell footprint on the surface because of viscoelastic wetting (13,14). Once on the surface, the cell must test for the presence of suitable ligands and then bind to them (15,16). This specific adhesion must occur for the cell to spread (17). Then, the cell tests the elasticity of the extracellular matrix (ECM), and on sufficiently stiff substrates, it continues spreading, approaching its maximal footprint area. Finally, after polarization is triggered on stiff substrates, the cell may start moving in a particular direction.

The dynamics of cells spreading has been studied extensively, and several characteristic universal features have been established (2,12,18–20). In particular, the average cell area has been shown to grow with time as a power law, often with the radius of cell footprint being $R \propto [t - \tau_{lag}]^{1/2}$, where the "lag" τ_{lag} is referred to as the adhesion time (18,21–23). It is important to note that the "lag time" is observed in many discussions of the dynamics of spreading but mostly ignored by subtracting it from the data. Several mechanistic models have been developed of how the cell spreading is achieved after the adhesion to ECM is established (18,21,23) as well as the spreading and cell orientation response to mechanical deformation of the substrate (24,25). A common theme to these studies is the presentation of individual cell trajectories, outlining the time course of a cell response to adhesion (although, of course, many cells are used to generate statistics). In contrast, here, we examine the dynamics of a cell population

Submitted May 4, 2018, and accepted for publication December 28, 2018.
*Correspondence: emt1000@cam.ac.uk
Editor: Stanislav Shvartsman.
https://doi.org/10.1016/j.bpj.2018.12.020
© 2019 Biophysical Society.
This is an open access article under the CC BY license (http://creativecommons.org/licenses/by/4.0/).

by identifying the time at which a cell reaches a specific point early in its spreading sequence (essentially reflecting the "decision" of a cell to start spreading in response to its ECM mechanosensing signal). Frisch and Thoumine (26) have shown that in the early stages of spreading, the cell takes a spherical-cap morphology, and when the increasing adhesion energy becomes similar to the cell cortical tension, the cell contact angle crosses from greater than 90° (representing the partial dewetting) to less than 90° (representing the partial wetting). Such a binary condition, asking whether an event has taken place by a certain time rather than what events are taking place over the course of time, allows the use of stochastic theory to interrogate the cell dynamics, extracting useful information about the underlying kinetics of spreading. In particular, we are able to form a better understanding of the "lag time" and also identify the rate-limiting energy barrier that controls the transition of cells from the initial nonspecific binding to the final strongly adhered and widely spreading regime. This is a useful complementary approach to single-cell measurements. We also emphasize that here, and in the rest of this article, we are discussing isolated cells on a substrate; when cells adhere to each other, their shape transitions are controlled by other mechanisms, based on cadherin and associated pathways (27).

While reporting and discussing the cell area increase on stiffer substrates, Fig. 5d of the article by Yeung et al. (2) and Fig. 2A of the article by Reinhart-King et al. (20) also present data on the time dependence of cell spreading, which already gives a hint for our central experimental finding: the onset of cell spreading does not depend on the substrate. In this article, we investigate the time dependence (kinetics) of the initiation of spreading, asking the following question: how long does it take for the cell to recognize the presence of a substrate and respond by engaging signaling pathways and enacting the required morphological change (spreading on the substrate)? Fig. 1 illustrates the point: plots (Fig. 1, *a* and *b*) show the same cells immediately after planting on the substrate and after some time when several cells have already responded by engaging their spreading. We plated two very different cell lines (National Institutes of Health (NIH)/3T3 fibroblasts and EA.hy927 endothelial cells) on a variety of substrates that span the range of stiffness from 30 GPa (stiff glass) to 460 Pa (very soft gel), registering the characteristic time at which the initially deposited planktonic cells start to spread.

We discover three things: 1) the onset of spreading is completely universal, not depending on the stiffness of substrates (in contrast to the final cell morphology, which strongly depends on it); 2) the rate-limiting process, with the characteristic free energy barrier, is the same in both cell lines; and 3) the onset of spreading is controlled by a nucleation event, its universal power-law dependence t^5, suggesting that there are five state changes a newly deposited cell must go through before it is able to spread. We also measure the sum of the free-energy changes of these state changes and find that this, in contrast to the rate-limiting process, depends on the cell line.

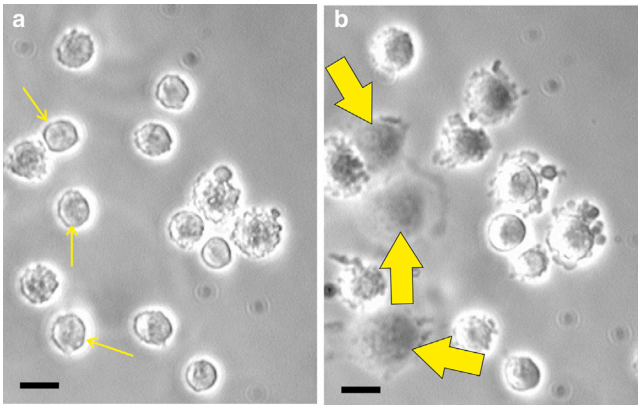

FIGURE 1 A section of the experimental field of view, illustrating the onset of spreading. Photographs (*a*) and (*b*) show the same cells immediately after planting on the substrate (*solid glass* with fibronectin) and 15 min later, when several cells have already responded by spreading (labeled by *matching arrows*). Scale bars, 20 μm. To see this figure in color, go online.

At first, our results on the insensitivity of the onset of spreading to substrate stiffness look counter to much of the literature. It is important to draw a clear line between many existing results on the cell area increase with time on different substrates, and our study looking at the statistics of a cell population that is starting to spread. In particular, the criterion we observe happens at a very early stage of the overall spreading (see Fig. S3), in which the cell area has increased only by a factor of 1.26 from its initial settled state.

MATERIALS AND METHODS

Cells and cell culture procedures

We chose to study endothelial cells and fibroblasts because their adhesion behavior is important for understanding cardiovascular diseases and tissue engineering, using immortalized cell lines: NIH/3T3 murine fibroblasts (obtained from American Type Culture Collection, Manassas, VA) and EA.hy927 endothelial cells.

NIH/3T3 fibroblasts are very well characterized because they have been used in many cell studies since their establishment as a cell line; they have also been used in cell adhesion studies, making them a good choice for our experiments (28,29). EA.hy927 is a cell line established in 1983 by the fusion of human umbilical vein endothelial cells with a lung carcinoma line (30). It has since become a widely used and thus well-characterized cell line, popular in studies of cardiovascular diseases. EA.hy927 cells have also been used for adhesion strength assays (31).

Cells were normally cultured at 37°C and 5% CO_2 in Dulbecco's modified Eagle's medium, from Greiner (Monroe, NC), with 10% fetal bovine serum and 1% penicillin/streptomycin, from Sigma-Aldrich (St. Louis, MO) (see Supporting Materials and Methods for detail). For a comparative study of the role of nutrients in the medium, we also used phosphate-buffered saline (PBS) from Thermo Fisher Scientific (Waltham, MA) during the spreading experiments.

Substrates of varying stiffness

To span a wide range of substrate stiffness, we used standard laboratory glass (elastic modulus 30 GPa) and several versions of siloxane elastomers: Sylgard 184 and Sylgard 527, the latter used with the compound/hardener ratio of 1:1 and 5:4. The resulting elastomers were tested on a standard laboratory rheometer (Anton Paar, Graz, Austria), giving the values of equilibrium modulus $G = 460$ Pa (for Syl527 5:4), 480 kPa (for Syl184), and 30 GPa for glass (zero-frequency limit shown in Fig. S1). For comparison, the stiffness of typical mammalian tissues is 100 Pa–1 kPa in brain tissue, ~3 kPa in adipose tissue, 10–20 kPa in muscle, 30–50 kPa in fibrous tissue, and up to a few MPa for bone. We avoided applying the commonly used plasma treatment because this was making the surface highly uneven on a micron scale, which would affect the adhesion. All surfaces were cleaned by ultrasonication in 96% ethanol for 15 min and then incubated with 10 μg/mL fibronectin in PBS for 45 min.

Experimental procedure and data acquisition

In our standard cell-spreading experiment, the cell culture dish was inserted into a closed chamber that maintained controlled temperature with an active water bath, and the CO_2 atmosphere, with microscope observation from the top. The cell culture (density 5×10^5 cells per mL, counted by the Neubauer chamber) was placed over the entire substrate. Cells were left to adhere to the substrate for 2 min, at which point the culture dish containing the substrate was filled slowly with fresh medium to reduce the cell density. This was to prevent new cells depositing and cell clusters forming on the substrate. Only the cells attached to the substrate at this point were included in the subsequent counting. This initial attachment is certainly purely physical through van der Waals forces and various nonspecific cell adhesion molecule headgroups. These physically adhered cells, initially spherical in planktonic culture, maintain the high spherical-cap shape with only a small adhesion footprint as ordinary inflated bilayer vesicles would do as well. This is readily confirmed by the optical interference bands around the cell perimeter and the lensing effect focusing the light by the short-focal distance near-spherical shape (see Supporting Materials and Methods, and also (26) for detail).

After a certain time on the substrate, the cells finally engage their specific adhesion-mechanosensing mechanism and start spreading, achieving a very widely spread area with highly asymmetric focal adhesions on stiff substrates or a round dome-like shape on soft substrates. We are looking to determine the time it takes for the cells to engage this active spreading process.

To obtain a population distribution of the onset time of cell spreading, we had to choose a spreading criterion that would be clear and easily distinguishable to avoid counting errors. We choose to count the initial onset of visible spreading, seen as the transition between the near-spherical cell initially planted (physically attached) on the substrate and the cell with adhesion processes engaged and its shape developing an inflection zone around the rim (see Fig. S2 for a more detailed illustration and explanation and Fig. S3 for an illustration in which this criterion is reached in the "standard" cell-spreading curves showing the area increase with time). This morphological transition turns out to be easily identified as the near-spherical cell has a sharp edge with interference bands in higher magnification and also a lensing effect of focusing light, which disappears in the transition to a more flattened shape. It must be emphasized that for our cell counting to be meaningful, the cells have to be isolated on the substrate; once the cells come into contact with each other, many other adhesion and mechanosensing mechanisms engage (for example, those based on cadherins), and they spread much more readily and more significantly. That is why our initial cell density was chosen so that the initial attachment is in isolation, and our spreading criterion is applied before they spread sufficiently to come in contact (as some cells in Fig. 1 have done).

We have carried out many dozens of such spreading experiments, deliberately varying the conditions: comparing cells of different generation and age (passage number), medium with and without penicillin/streptomycin, with and without CO_2 tent, and at slightly varying pH of the medium—all on different substrates and at different temperatures. Fig. S3 illustrates the robustness and reproducibility of these experiments, which also confirms the meaningful use of the "spreading criterion."

In each individual experiment (given substrate, fixed temperature, and other parameters), once the cells were deposited on the substrate and the clock started, we took broad-field microscopic images at regular time intervals and counted the fraction of cells that have crossed the threshold defined by our spreading criterion—that is, the cells that have started the active spreading process in response to their mechanosensing cue. This produced a characteristic sigmoidal curve for each experiment (see Fig. 2); the fraction of cells engaged in spreading starting from zero at $t = 0$ and saturating at near 100% at a very long time (if we

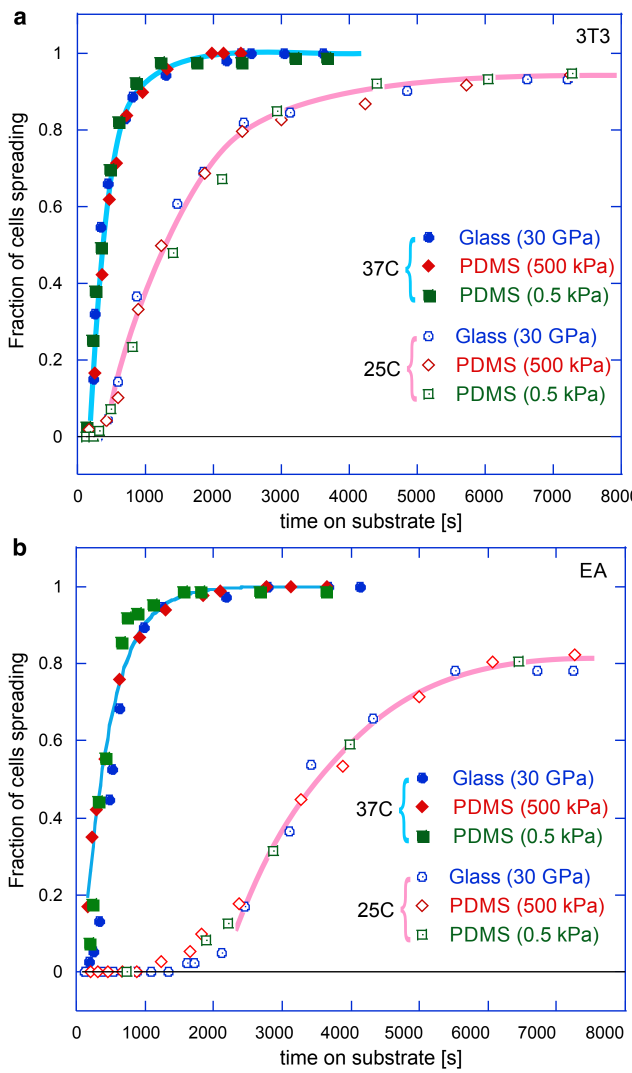

FIGURE 2 Cumulative population dynamics of cell spreading. Plots (*a*) and (*b*) show the growing fraction of cells engaged in spreading on substrates with different stiffness for 3T3 fibroblasts and EA endothelial cells at two different temperatures each. It is clear that the dynamics is not affected by the substrate stiffness but is affected by changes with temperature. In the remainder of this article, we analyze in detail the long-time behavior of these cumulative curves as they approach saturation, and the behavior at short times when the onset of mechanosensing response occurs. To see this figure in color, go online.

exclude the occasional cell mortality, which was more of a factor at lower temperatures). The typical sample size was 100–120 cells in each experiment (field of view); however, we have taken many similar samples and verified the high fidelity of data. The main sources of error were inconsistency of application of the spreading criterion in image analysis, imperfections of fibronectin coverage on substrate, temperature fluctuations, and, of course, the natural cell variability. All of these are random errors with no systematic drift. We were satisfied that the results were reproducible, and errors did not dominate the data trends. The plots in Figs. 2 and 3 do not include error bars not to obscure distinct data sets, but the reader could gauge this error from Fig. S3.

RESULTS

We first emphasize that our experiments concurred with the results of earlier studies (1,2,4,26). Cells placed on stiffer substrates spread to larger areas and were less rounded for both our cell types. There is also a strong dependence on the ECM protein coverage (32), but this was not a variable in our study.

The time of initiation of spreading is presented in Fig. 2. These two plots (for 3T3 and EA cells) show the fraction of cells that have started spreading at each given time that has passed after planting on substrates and replacing the medium. The point of steepest gradient in these cumulative curves marks the most probable time for the onset of spreading (see Supporting Materials and Methods for the detailed analysis). We see the timing of cell spreading is completely insensitive to the substrate stiffness; the kinetics of a spreading response is exactly the same on each substrate. The work of Margadant et al. (33) has reported a similar effect (the rate of spreading did not depend on the degree of ECM protein coverage on the surface). Instead of substrate stiffness, we find the curves in Fig. 2 are strongly segregated by temperature.

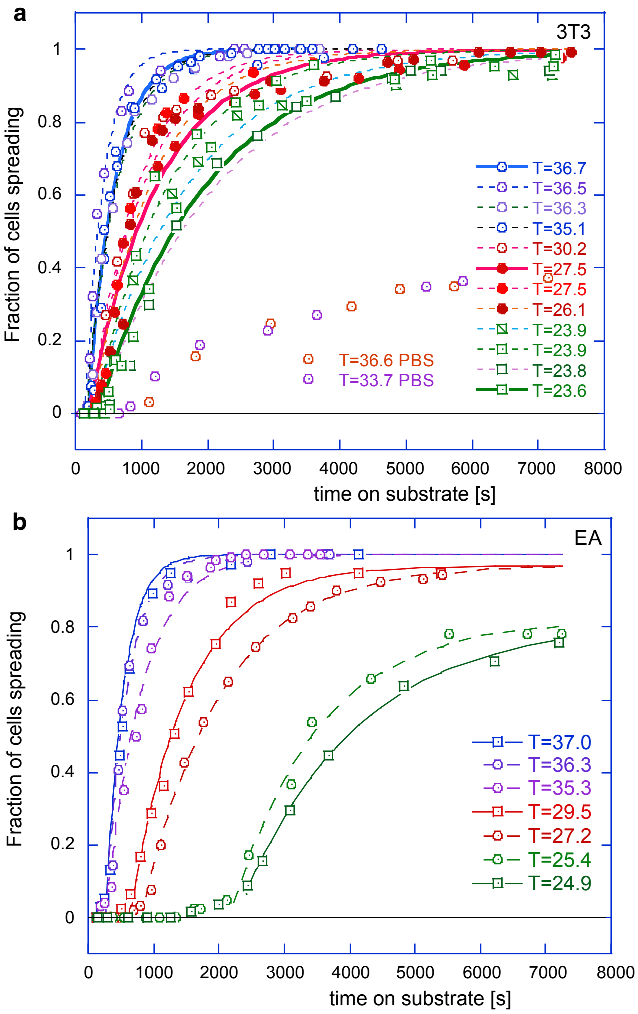

FIGURE 3 Cumulative population dynamics of cell spreading. Plots (*a*) and (*b*) show fraction of spreading cells on glass at many different temperatures for 3T3 fibroblasts and EA endothelial cells. Lines in all plots are the fits of the long-time portion of data with the exponential relaxation curves, producing the fitted values of the longest relaxation time τ (see text). To see this figure in color, go online.

Long-time trend: A rate-limiting process

To examine the effect of temperature in greater detail, in Fig. 3, we plotted the same cumulative spreading fraction curves for the two cell types on glass (as we are now assured that these curves are the same on all substrates). It is noticeable that the initial lag is greater in the EA cells and that at low temperature, the saturation level drops significantly below 100%—presumably because more cells disengage (or die) at low temperatures, reducing the saturation fraction. The same effect is much enhanced for the nutrient-starved cells in the PBS medium (see in Fig. 3 *a*); the onset of spreading is very slow in this case, and a large fraction of cells do not engage at all. Nevertheless, the generic sigmoidal shape of these cumulative curves is universal, and the random spread of data within each individual experiment is not excessive. We then look to analyze the trends in this time dependence.

The curves of the generic shape seen in Figs. 2 and 3 are encountered in many areas of science, and their characteristic foot at early times, especially obvious at lower temperatures, is usually associated with a lag in the corresponding process. We will discuss this early-time regime separately, later in the article, but first, we fit exponential relaxation curves to the long-time portion of the data (as the *fit lines* in Fig. 3 indicate): $Q(t) = A \times (1 - \exp[-(t - t_{lag})/\tau]$. The Supporting Materials and Methods give the table of values of A and τ for each curve, but it is clear from the plots that the fitting to the single-exponential relaxation law, with just two parameters because A is known for each curve, is very successful. The characteristic relaxation time τ markedly increases at low temperatures. It is interesting that such a characteristic time associated with the "spreading of an average cell" has been discussed in (18), giving the same order of magnitude (of the order of magnitude 50–100 s).

To better understand this dependence on temperature, we tested a hypothesis that this relaxation time is determined by the thermally activated law by producing the characteristic

Arrhenius plots of relaxation times for both cell types (see Fig. 4). It is remarkable that both cells show almost exactly the same trend of their relaxation time. The rate-limiting process in their spreading pathways is the same: $\tau = \tau_0 e^{\Delta G/k_B T}$, with the activation energy $\Delta G \approx 18.3 \pm 1.5$ kcal/mol and the thermal rate of attempts $\tau_0^{-1} \approx 4 \times 10^{10}\ \mathrm{s}^{-1}$. Both values are very sensible; this magnitude of ΔG is typical for the noncovalent bonding energy between protein domains (34), and this rate of thermal collisions is in excellent agreement with the basic Brownian motion values.

Early-time dynamics

After discovering that the late-time (rate-limiting) dynamics of the onset of spreading is quite universal across different cells and substrates, it becomes clear that the marked difference between the two cell lines in Fig. 3 lies in the early-time behavior, something that we have called a "lag" after many similar situations in protein self-assembly. To examine this early-time regime more carefully, we replotted the same time series data on the log-log scale in Fig. 5.

This reveals that the process is active from the very beginning ($t = 0$), and the plotted value grows as a power law of time. The only reason that we appear to see a "lag" is because our experimental technique of counting the cells engaging in spreading did not permit values below 0.01 (1%) to be resolved in this plot; the same certainly applies to other experimental situations reporting similar kinetic data. The trend illustrated in Fig. 5 is clear; the early onset of cell spreading follows a universal power law, and the fitting of all our data sets gives $Q(t) = \alpha t^5$ with very good accuracy, where only the prefactor α depends on temperature and the cell type. We find this result truly remarkable: similar to the universal value of binding energy that controls thermally activated rate-limiting relaxation time τ, this very specific t^5

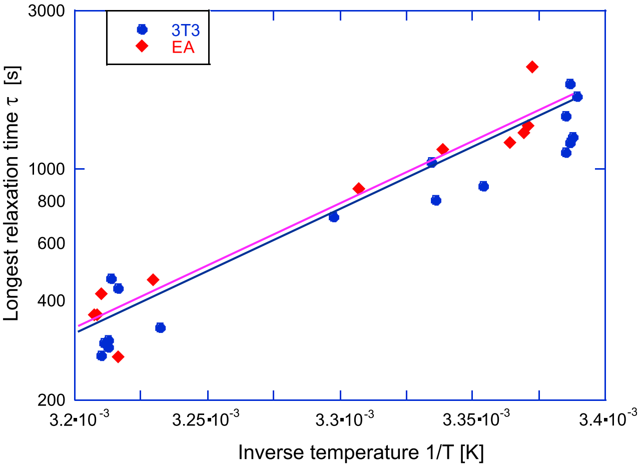

FIGURE 4 The Arrhenius plot of the longest relaxation time (log(τ) versus inverse absolute temperature) from the exponential fits in Fig. 3, *a* and *b*, giving almost exactly the same value of binding energy $\Delta G \approx$ 18 kcal/mol for both types of cells. To see this figure in color, go online.

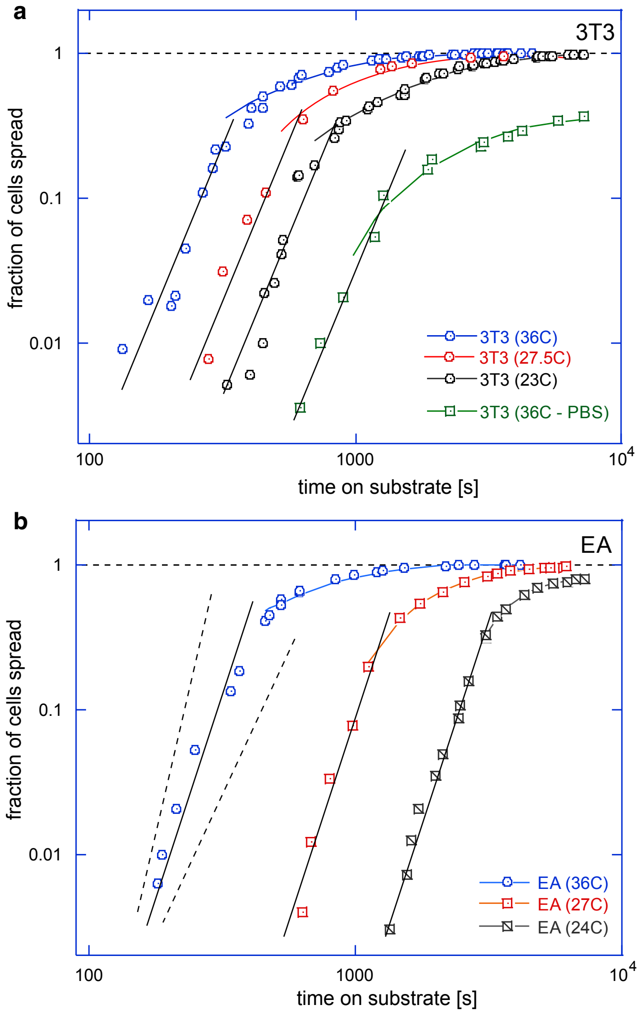

FIGURE 5 Analysis of the short-time dynamics of cell spreading. Plots (*a*) and (*b*) show selected data sets from Fig. 3, *a* and *b* presented on the log-log scale to enhance the short-time dynamical range. In both plots, the power-law slopes of the short-time data follow the equation αt^5, with the coefficient prefactor α depending both on cell type and on temperature. The dashed line illustrates the slopes of t^6 and t^4 to illustrate the strength of fit. To see this figure in color, go online.

power law appears to be the only sensible fit of the early-time data for different cells, temperatures, and substrates.

Again, strong temperature dependence is evident in the subpopulations of cells that start spreading very early; the difference was evident in Figs. 2 and 3 but is very clearly enhanced in Fig. 5. What changes between the data sets is the prefactor α of the universal power law αt^5, which has a systematic temperature dependence (the fitted values of $\alpha(T)$ are listed in Table S2). Now expecting the thermally activated behavior, by analogy with the earlier analysis, we plot these prefactors $\alpha(T)$ on the Arrhenius plot in Fig. 6. The fitting to $\alpha = \mathrm{const} \times e^{-\Delta H/k_B T}$ indeed gives a very reasonable trend with the activation energies $\Delta H =$ 70 kcal/mol for 3T3 and 129 kcal/mol for EA. Note that, in contrast to Fig. 4, here, we have a negative exponent

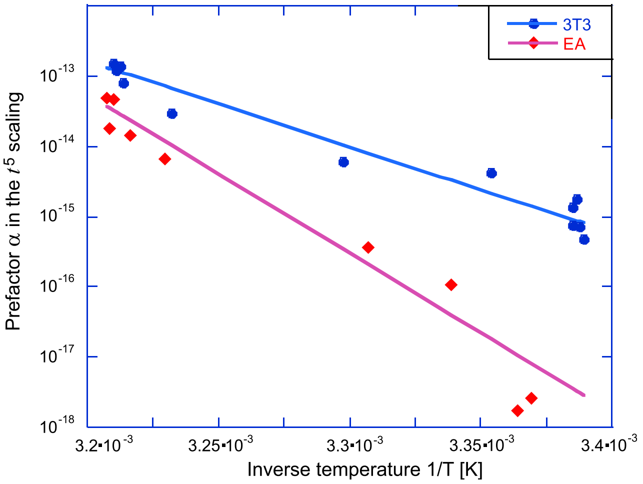

FIGURE 6 Analysis of the short-time dynamics of cell spreading. The Arrhenius plot of the prefactor $\alpha(T)$, with the fit lines giving the effective activation enthalpy $\Delta H \approx 70$ kcal/mol for 3T3 and 129 kcal/mol for EA. See text, which explains how this value represents the sum of free energy barriers of key proteins assembling into the adhesion complex. To see this figure in color, go online.

(i.e., the parameter $\alpha(T)$), which represents a reaction rate rather than a relaxation time. In the classical Arrhenius-Kramers thermal activation, the process time is shorter as the temperature increases, whereas Fig. 6 shows the scaling factor $\alpha(T)$ is decreasing as the temperature decreases instead (which is reflected in the overall observation of longer lag time in the cumulative curves).

DISCUSSION

The time we measure is the sum of the adhesion lag time and the time to reach our binary criterion for the start of spreading. The distribution in our data is due to the statistical distribution of the "lag times." Our results show that the stochasticity of lag time has structure. We can use this structure to infer information about the processes underlying adhesion and spreading.

In classical physics, early-time power-law kinetics in a cumulative distribution are a hallmark of self-assembly processes, such as polymerization or aggregation (35,36). The reason for this is that at short times, the kinetics are dictated by the number of states you must pass through to reach a final state. In this case, we must be looking at a process of self-assembly within the cell. The exponent of the power law gives us some idea of how many important assembly steps there are. But, what exactly are we assembling? To us, it seems likely that we are observing the formation of adhesion points and adhesome complexes that allow the cell to bind onto its ECM environment and begin spreading. The idea is that the initial assembly of adhesome complexes is responsible for the initial changes in the cell footprint area (e.g., Reinhart-King et al. (20)). Here, we are able to infer some quantitative details of this process.

It is well established that disruption of the integrin-fibronectin linkage completely halts cell spreading (17,37). Integrins are transmembrane receptors linking the cell to the matrix in focal adhesions (38–40). To attach to their ligands, they need to be activated (41,42); in isolation, integrin pairs will lie in their inactive state, unable to bind to fibronectin (or other ECM proteins containing the RGD motif). In equilibrium, the level of integrin activation might be dependent on the ECM rigidity (43,44); however, here, we are examining very early stages of cell settling on its substrate, so it is the adhesome assembly and signaling that control our results.

Much of the literature on focal adhesions sees the attachment of the talin head domain to integrin tails as an important activation step (45–47). Talin is a key protein in mature and nascent adhesions, linking integrins to the actin cytoskeleton and providing a scaffold for other focal adhesion proteins (see, for example, (48)). For the onset of spreading, there is some conflict in the literature; in the study by Zhang et al. (17), in which they confirmed that integrin linkage was essential to the onset of spreading, they actually depleted both types of talin and found that the onset of spreading was not fully inhibited, although spreading was severely limited. This could indicate that talin was not needed for the activation of integrins during the onset of spreading. However, a subsequent knockout study of talin (among other proteins) (49) found that spreading was actually completely inhibited by the removal of talin (although partial function was restored by the addition of Mn^{2+}). In that work, the authors note that the experimental methods (small interfering RNA transfection) employed in previous studies left residual amounts of proteins in the cell and that there may well have been enough talin left in depleted cells to form nascent adhesions. Indeed, in their article, Zhang et al. say that the decrease in talin2 levels (talin1 was not expressed in their cell lines) was between 40 and 68%.

In fact, Theodosiou et al. (49) implicate three further players: kindlins, paxillin, and focal adhesion kinase (FAK). This is not a new finding or point of view; since the early discovery of the key role of FAK in the integrin adhesome (40,50,51), it was understood that is the FAK activation that produces the chemical cue for the subsequent cell mechanosensing pathways via Src, Rho, Rac, and Cdc42 as well as Erk (37,52–54). Theodosiou et al. found that chemical inhibition of FAK reduced lamellopodia formation in cells to the level of kindlin knockout cells (49). The formation of these lamellopodia and the initiation of isotropic cell spreading was therefore found to be dependent on FAK activation. A recent model of FAK as a mechanosensor (55) shows how the rate of its activation is sensitive to the stiffness of substrate and the cytoskeletal pulling force. Importantly, when the force is low (as we would expect at early times before the mechanosensing pathways are activated and the cytoskeletal forces increase), this rate is controlled only by the bonding energy between its FERM and kinase domains, not the stiffness.

FAK clearly sits at the center of the adhesion signaling network (56). But the minimal composition of the whole adhesion-mechanosensing complex in the nascent adhesions as well as the rate of its assembly and turnover remain a question of active research and debate. Kindlins are known to be a necessary partner for talin in integrin activation (41,47,48). The F3 subdomain of a FERM domain mediates an interaction with β-integrin tails and "cooperates" with the talin head domain in integrin activation (57). Paxillin is another player in the adhesion network (48,49,56). In particular, in the nascent adhesions formed at the onset of spreading, kindlin was directly binding paxillin; paxillin was then recruiting FAK to these nascent adhesions. On the other hand, the important role of vinculin in several processes in the integrin-talin-FAK adhesion complex appears to be relevant mostly at the mature focal adhesion stage (33,58,59), and we believe its role is to bind different adhesion complexes into a dense focal adhesion raft.

How does this information tie in with our results? A recent molecular dynamics simulation (34) has explicitly calculated the bonding energy between FERM and kinase domains of FAK as $\Delta G \approx 17$ kcal/mol. Breaking this bond is the essential step of FAK activation. If we associate this barrier with the longest relaxation time examined in Fig. 4, the agreement of the ΔG values is remarkably close. According to the reaction rate theory, this energy barrier is the largest one of the assembly process because it produces the long-time "bottleneck" in the population dynamics of the onset of spreading.

In a scenario in which the spreading response is initiated by the assembly of adhesome complex and the engagement of mechanosensors, the cell must undergo five changes of state before it can start spreading, with the last being the FAK activation process (55) (see Supporting Materials and Methods for detail). This is necessary for the mechanosensing signal to be generated and the cell morphological response initiated (48); it also has to be the rate-limiting step, logistically. The possible candidates for the other four reaction steps must have a rate slow enough to be counted in the first data points (see Fig. 7 for an illustration). Images of cells were taken approximately every

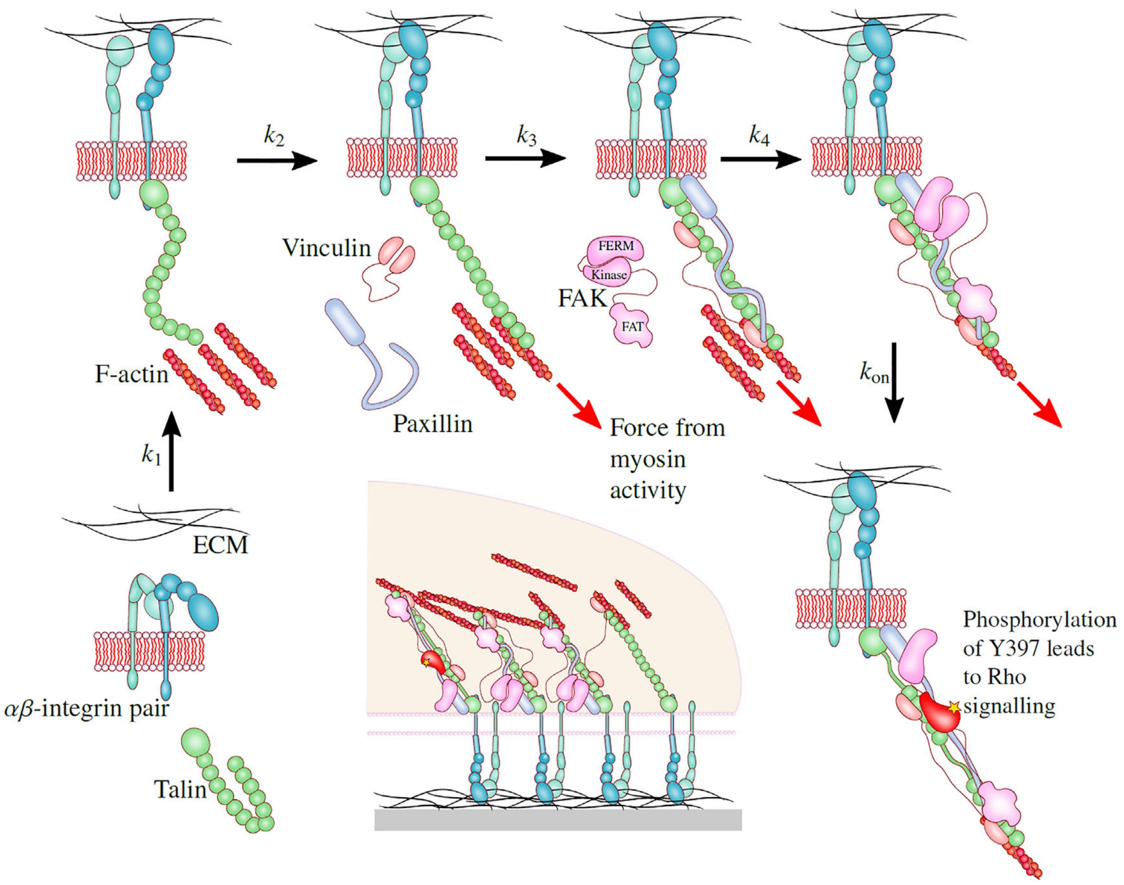

FIGURE 7 A possible assembly sequence of a mechanosensor complex. Our analysis suggests that there are five distinct slow stages illustrated in the sequence, with their respective rates $k_1 - k_4$ and the rate of FAK activation k_{on} (controlled by the free energy barrier $\Delta G \approx 18$ kcal/mol, cf. Fig. 4). The product of the five rate constants $\alpha = k_1, k_2, k_3, k_4$, and k_{on} is what we measure in the Arrhenius plot in Fig. 6. In the center is a sketch of forming focal adhesion cluster, in which the individual mechanosensor complexes in various stages of development/turnover are bound by vinculin and actin cross-linking (see text for details and references). To see this figure in color, go online.

minute, and so it is impossible to resolve fast processes with rates of $k > 1\ \mathrm{min}^{-1}$ using our data. For instance, the binding of integrins to fibronectin does not fit this criterion. It has been seen that the binding of integrins to an antibody ligand in the presence of different cations has a characteristic binding time of 0.01–1 ms (60); this is much faster than we could resolve in our experimental data. To form the force-bearing chain from integrin to F-actin of cytoskeleton, we see the following reactions necessary: 1) the binding of talin and kindlin to integrins, 2) the binding of paxillin to kindlin, 3) the binding of talin to F-actin, 4) the binding of FERM domain of FAK to talin, 5) the binding of FAT domain of FAK to paxillin, and 6) the binding of FAK/paxillin to the F-actin. It is difficult to find any estimates of the rates of these processes. One can find evidence for the fast strengthening of focal adhesions under load (61), but this is not the same as the assembly of these complexes at the onset of spreading. Our experiments suggest that four of these reactions are quite slow (accounting for the need of protein localization on the complex); we cannot be certain which, but we have measured the combined activation energy of these four reactions (Fig. 6) in 3T3 and EA cells. Only once the full force chain of the integrin adhesome is assembled can the mechanosensor produce the signal for the cell to modify its morphology to the substrate.

Another possible scenario that could account for our five-step initial kinetics still has to rely on activation and adhesion of integrins but could include a phase of initial viscoelastic spreading (18) that should be controlled by physical interactions on a more macroscopic scale. In that case, we would require a few slow steps of adhesome assembly. We cannot rule this possibility out with our data, but it is interesting to note that the universal timescale suggested by Cuvelier et al. (obtained with a much lower ligand density; fibronectin coating 10 times less dense as ours) was between 5 and 10 min. Using their model with parameters they fitted for HeLa cells with our fibronectin density, gives the estimate of a spreading time to our criterion of around 2–3 min. As such, this is not inconsistent with our data, with the caveat that we are still seeing the adhesion process before spreading in the early power-law kinetics. It is also unclear whether there should be an Arrhenius activation-type temperature dependence for their spreading timescale (which is prominent in our data). Certainly, the work of Cuvelier et al. avoids kinetic complications by simply considering the adhesion energy gain per unit area of the cell.

The unusual feature of this work is the use of population dynamics of spreading cells to infer details of the microscopic processes governing the cell response to an external substrate. By linking the results to nucleation theory, details of which are given in Supporting Materials and Methods, we found a, to our knowledge, novel way of looking at the onset of cell spreading as a problem of complex assembly.

SUPPORTING MATERIAL

Supporting Materials and Methods, six figures, and two tables are available at http://www.biophysj.org/biophysj/supplemental/S0006-3495(19)30015-3.

AUTHOR CONTRIBUTION

A.-L.R. carried out all experiments. S.B., A.-L.R., and E.M.T. carried out different elements of data analysis. S.B. and E.M.T. wrote the article.

ACKNOWLEDGMENTS

The authors acknowledge many helpful discussions with K. Franze, K. Chalut, A. B. Kolomeisky, and H. Welch. The comments of U. S. Schwarz and the experimental support in the cell culture lab by E. Nugent and F. Morgan are much appreciated. This work has been funded by Engineering and Physical Sciences Research Council (grants EP/M508007/1 and EP/J017639) and the Ernest Oppenheimer Trust in Cambridge.

REFERENCES

1. Discher, D. E., P. Janmey, and Y. L. Wang. 2005. Tissue cells feel and respond to the stiffness of their substrate. *Science.* 310:1139–1143.
2. Yeung, T., P. C. Georges, …, P. A. Janmey. 2005. Effects of substrate stiffness on cell morphology, cytoskeletal structure, and adhesion. *Cell Motil. Cytoskeleton.* 60:24–34.
3. Nisenholz, N., K. Rajendran, …, A. Zemel. 2014. Active mechanics and dynamics of cell spreading on elastic substrates. *Soft Matter.* 10:7234–7246.
4. Engler, A. J., S. Sen, …, D. E. Discher. 2006. Matrix elasticity directs stem cell lineage specification. *Cell.* 126:677–689.
5. Hinz, B. 2007. Formation and function of the myofibroblast during tissue repair. *J. Invest. Dermatol.* 127:526–537.
6. Tomasek, J. J., G. Gabbiani, …, R. A. Brown. 2002. Myofibroblasts and mechano-regulation of connective tissue remodelling. *Nat. Rev. Mol. Cell Biol.* 3:349–363.
7. Solon, J., I. Levental, …, P. A. Janmey. 2007. Fibroblast adaptation and stiffness matching to soft elastic substrates. *Biophys. J.* 93:4453–4461.
8. Sinha, S., M. H. Hoofnagle, …, G. K. Owens. 2004. Transforming growth factor-β1 signaling contributes to development of smooth muscle cells from embryonic stem cells. *Am. J. Physiol. Cell Physiol.* 287:C1560–C1568.
9. Cheung, C., A. S. Bernardo, …, S. Sinha. 2012. Generation of human vascular smooth muscle subtypes provides insight into embryological origin-dependent disease susceptibility. *Nat. Biotechnol.* 30:165–173.
10. Alliston, T., L. Choy, …, R. Derynck. 2001. TGF-β-induced repression of CBFA1 by Smad3 decreases cbfa1 and osteocalcin expression and inhibits osteoblast differentiation. *EMBO J.* 20:2254–2272.
11. Butcher, D. T., T. Alliston, and V. M. Weaver. 2009. A tense situation: forcing tumour progression. *Nat. Rev. Cancer.* 9:108–122.
12. Schwarz, U. S., and S. A. Safran. 2013. Physics of adherent cells. *Rev. Mod. Phys.* 45:1327–1381.
13. Bruinsma, R., and E. Sackmann. 2001. Bioadhesion and the dewetting transition. *C. R. Acad. Sci. Paris.* 2:801–815.
14. Sackmann, E., and R. F. Bruinsma. 2002. Cell adhesion as wetting transition? *Chemphyschem.* 3:262–269.
15. Döbereiner, H. G., B. J. Dubin-Thaler, …, M. P. Sheetz. 2005. Force sensing and generation in cell phases: analyses of complex functions. *J. Appl. Physiol.* 98:1542–1546.
16. Cohen, M., D. Joester, …, L. Addadi. 2004. Spatial and temporal sequence of events in cell adhesion: from molecular recognition to focal adhesion assembly. *Chembiochem.* 5:1393–1399.

17. Zhang, X., G. Jiang, …, M. P. Sheetz. 2008. Talin depletion reveals independence of initial cell spreading from integrin activation and traction. *Nat. Cell Biol.* 10:1062–1068.

18. Cuvelier, D., M. Théry, …, L. Mahadevan. 2007. The universal dynamics of cell spreading. *Curr. Biol.* 17:694–699.

19. Brill-Karniely, Y., N. Nisenholz, …, A. Zemel. 2014. Dynamics of cell area and force during spreading. *Biophys. J.* 107:L37–L40.

20. Reinhart-King, C. A., M. Dembo, and D. A. Hammer. 2005. The dynamics and mechanics of endothelial cell spreading. *Biophys. J.* 89:676–689.

21. Li, J., D. Han, and Y. P. Zhao. 2014. Kinetic behaviour of the cells touching substrate: the interfacial stiffness guides cell spreading. *Sci. Rep.* 4:3910.

22. Döbereiner, H. G., B. Dubin-Thaler, …, M. P. Sheetz. 2004. Dynamic phase transitions in cell spreading. *Phys. Rev. Lett.* 93:108105.

23. Xiong, Y., P. Rangamani, …, R. Iyengar. 2010. Mechanisms controlling cell size and shape during isotropic cell spreading. *Biophys. J.* 98:2136–2146.

24. Xu, G. K., B. Li, …, H. Gao. 2016. A tensegrity model of cell reorientation on cyclically stretched substrates. *Biophys. J.* 111:1478–1486.

25. Xu, G. K., X. Q. Feng, and H. Gao. 2018. Orientations of cells on compliant substrates under biaxial stretches: a theoretical study. *Biophys. J.* 114:701–710.

26. Frisch, T., and O. Thoumine. 2002. Predicting the kinetics of cell spreading. *J. Biomech.* 35:1137–1141.

27. Buckley, C. D., J. Tan, …, A. R. Dunn. 2014. Cell adhesion. The minimal cadherin-catenin complex binds to actin filaments under force. *Science.* 346:1254211.

28. Todaro, G. J., and H. Green. 1963. Quantitative studies of the growth of mouse embryo cells in culture and their development into established lines. *J. Cell Biol.* 17:299–313.

29. Rocha, A., M. Hahn, and H. Liang. 2010. Critical fluid shear stress analysis for cell–polymer adhesion. *J. Mater. Sci.* 45:811–817.

30. Edgell, C. J., C. C. McDonald, and J. B. Graham. 1983. Permanent cell line expressing human factor VIII-related antigen established by hybridization. *Proc. Natl. Acad. Sci. USA.* 80:3734–3737.

31. Han, Y. L., Q. Xu, …, J. Y. Wang. 2013. Cell adhesion on zein films under shear stress field. *Colloids Surf. B Biointerfaces.* 111:479–485.

32. Dubin-Thaler, B. J., G. Giannone, …, M. P. Sheetz. 2004. Nanometer analysis of cell spreading on matrix-coated surfaces reveals two distinct cell states and STEPs. *Biophys. J.* 86:1794–1806.

33. Margadant, F., L. L. Chew, …, M. Sheetz. 2011. Mechanotransduction in vivo by repeated talin stretch-relaxation events depends upon vinculin. *PLoS Biol.* 9:e1001223.

34. Zhou, J., C. Aponte-Santamaría, …, F. Gräter. 2015. Mechanism of focal adhesion kinase mechanosensing. *PLoS Comput. Biol.* 11:e1004593.

35. Hofrichter, J., P. D. Ross, and W. A. Eaton. 1974. Kinetics and mechanism of deoxyhemoglobin S gelation: a new approach to understanding sickle cell disease. *Proc. Natl. Acad. Sci. USA.* 71:4864–4868.

36. Valleriani, A., X. Li, and A. B. Kolomeisky. 2014. Unveiling the hidden structure of complex stochastic biochemical networks. *J. Chem. Phys.* 140:064101.

37. Price, L. S., J. Leng, …, G. M. Bokoch. 1998. Activation of Rac and Cdc42 by integrins mediates cell spreading. *Mol. Biol. Cell.* 9:1863–1871.

38. Hynes, R. O. 2002. Integrins: bidirectional, allosteric signaling machines. *Cell.* 110:673–687.

39. Giancotti, F. G. 2000. Complexity and specificity of integrin signalling. *Nat. Cell Biol.* 2:E13–E14.

40. Guan, J. L., J. E. Trevithick, and R. O. Hynes. 1991. Fibronectin/integrin interaction induces tyrosine phosphorylation of a 120-kDa protein. *Cell Regul.* 2:951–964.

41. Kim, C., F. Ye, and M. H. Ginsberg. 2011. Regulation of integrin activation. *Annu. Rev. Cell Dev. Biol.* 27:321–345.

42. Shattil, S. J., C. Kim, and M. H. Ginsberg. 2010. The final steps of integrin activation: the end game. *Nat. Rev. Mol. Cell Biol.* 11:288–300.

43. Schwarz, U. S., T. Erdmann, and I. B. Bischofs. 2006. Focal adhesions as mechanosensors: the two-spring model. *Biosystems.* 83:225–232.

44. Xu, G. K., C. Yang, …, X. Q. Feng. 2014. Integrin activation and internalization mediated by extracellular matrix elasticity: a biomechanical model. *J. Biomech.* 47:1479–1484.

45. Tadokoro, S., S. J. Shattil, …, D. A. Calderwood. 2003. Talin binding to integrin β tails: a final common step in integrin activation. *Science.* 302:103–106.

46. Wegener, K. L., A. W. Partridge, …, I. D. Campbell. 2007. Structural basis of integrin activation by talin. *Cell.* 128:171–182.

47. Moser, M., K. R. Legate, …, R. Fässler. 2009. The tail of integrins, talin, and kindlins. *Science.* 324:895–899.

48. Geiger, B., J. P. Spatz, and A. D. Bershadsky. 2009. Environmental sensing through focal adhesions. *Nat. Rev. Mol. Cell Biol.* 10:21–33.

49. Theodosiou, M., M. Widmaier, …, R. Fässler. 2016. Kindlin-2 cooperates with talin to activate integrins and induces cell spreading by directly binding paxillin. *eLife.* 5:e10130.

50. Sieg, D. J., C. R. Hauck, …, D. D. Schlaepfer. 2000. FAK integrates growth-factor and integrin signals to promote cell migration. *Nat. Cell Biol.* 2:249–256.

51. Parsons, J. T. 2003. Focal adhesion kinase: the first ten years. *J. Cell Sci.* 116:1409–1416.

52. Huveneers, S., and E. H. Danen. 2009. Adhesion signaling - crosstalk between integrins, Src and Rho. *J. Cell Sci.* 122:1059–1069.

53. Schwartz, M. A., and S. J. Shattil. 2000. Signaling networks linking integrins and rho family GTPases. *Trends Biochem. Sci.* 25:388–391.

54. Pajic, M., D. Herrmann, …, P. Timpson. 2015. The dynamics of Rho GTPase signaling and implications for targeting cancer and the tumor microenvironment. *Small GTPases.* 6:123–133.

55. Bell, S., and E. M. Terentjev. 2017. Focal adhesion kinase: the reversible molecular mechanosensor. *Biophys. J.* 112:2439–2450.

56. Zaidel-Bar, R., S. Itzkovitz, …, B. Geiger. 2007. Functional atlas of the integrin adhesome. *Nat. Cell Biol.* 9:858–867.

57. Moser, M., B. Nieswandt, …, R. Fässler. 2008. Kindlin-3 is essential for integrin activation and platelet aggregation. *Nat. Med.* 14:325–330.

58. Hemmings, L., D. J. Rees, …, D. R. Critchley. 1996. Talin contains three actin-binding sites each of which is adjacent to a vinculin-binding site. *J. Cell Sci.* 109:2715–2726.

59. Yao, M., B. T. Goult, …, J. Yan. 2014. Mechanical activation of vinculin binding to talin locks talin in an unfolded conformation. *Sci. Rep.* 4:4610.

60. Hu, D. D., C. F. Barbas, and J. W. Smith. 1996. An allosteric Ca2+ binding site on the β3-integrins that regulates the dissociation rate for RGD ligands. *J. Biol. Chem.* 271:21745–21751.

61. Strohmeyer, N., M. Bharadwaj, …, D. J. Müller. 2017. Fibronectin-bound $\alpha 5\beta 1$ integrins sense load and signal to reinforce adhesion in less than a second. *Nat. Mater.* 16:1262–1270.

Biophysical Journal, Volume *116*

Supplemental Information

Universal Kinetics of the Onset of Cell Spreading on Substrates of Different Stiffness

Samuel Bell, Anna-Lena Redmann, and Eugene M. Terentjev

Universal kinetics of the onset of cell spreading: Supplementary Information

Samuel Bell, Anna-Lena Redmann and Eugene M. Terentjev[1]
Cavendish Laboratory, University of Cambridge, Cambridge, CB3 0HE, U.K.

Cells and cell culture procedures

There are different types of endothelial and fibroblast cells available. Primary cells are directly taken from donor tissue and then grown in cell culture conditions. They can be grown in culture for a specific amount of time before they undergo senescence and die. The advantage of primary cells is that they are as close to in-vivo cells as possible, but as they are taken from different donors, their behaviour is less reproducible. Immortalized cell lines are obtained from primary cells by, for example, transfection or fusion. This results in a change in their DNA, leading to indefinite proliferation. This makes it easier to handle them in multiple long-term experiments, and makes such experiments more reproducible, but at the same time many immortalized cells have some tumorous behavior[1,2]. We used immortalized cell lines: NIH/3T3 murine fibroblasts (obtained from ATCC) and EA.hy927 endothelial cells.

Cells were normally cultured at 37C and 5% CO2 in Dulbecco's modified Eagle's medium (DMEM), from Greiner, with 10% fetal bovine serum and 1% Pen/Strep (solution stabilized, with 10,000 units penicillin and 10 mg streptomycin/mL), from Sigma Aldrich. For a comparative study of the role of nutrient in the medium during the spreading experiments, we also used phosphate-buffered saline (PBS), from Thermo Fisher Scientific. Cells were subcultured in DMEM every 3 days, at about 70% confluency, by trypsinization, to avoid the formation of big lumps of cells, thus ensuring that we maintain a single cell suspension. Cells were trypsinized for 5 min (Trypsin-EDTA 0.05%). The solution was then neutralized by added complete growth medium and centrifuged at 1000 rpm for 5 min. We tested our results on several parallel cell cultures that did not use Pen/Strep, and confirmed no significant difference in our results.

The use of Pen-Strep can be questioned. Antibiotics have been used prophylactically to prevent bacterial infections in cell culture for many years, and they are still being used. It was the introduction of antibiotics that allowed the widespread development of cell culture methods in the first place, as bacterial contamination was a major problem[3]. However, although toxicity experiments found that antibiotics were harmless to mammalian cells[4], there are concerns about the use of antibiotics in cell culture associated with a neglect of aseptic technique and possible side effects of antibiotics. Many adhesion strength studies use Pen/Strep or other antimycotic or antibiotic solutions in the cell culture, and we followed this procedure as well. We have tested our results on several parallel cell cultures that did not use Pen/Strep, and confirmed that no significant difference was inflicted on our results.

Substrates of varying stiffness

The plot in Fig. S1 shows the data from a standard frequency-sweep rheology test, in an oscillating parallel-plate rheometer (Anton Paar) at constant temperature of 25°C and low shear strain of 3% (after separately testing that this level of strain remains well within the linear-elastic regime). The frequency sweep shows the low-frequency rubber plateau of the storage modulus $G'(\omega)$, and an onset of the viscoelastic rise for the weaker gels. The usual dissipation peak, corresponding to the Rouse frequency of the average network strand, is expected to occur at 10-100 kHz. Importantly for us, the limit of equilibrium shear modulus is unambiguously reached in such a test. The values of the corresponding Young modulus are labelled in brackets for each curve (it is strictly three times the measured shear modulus because these elastomers are incompressible).

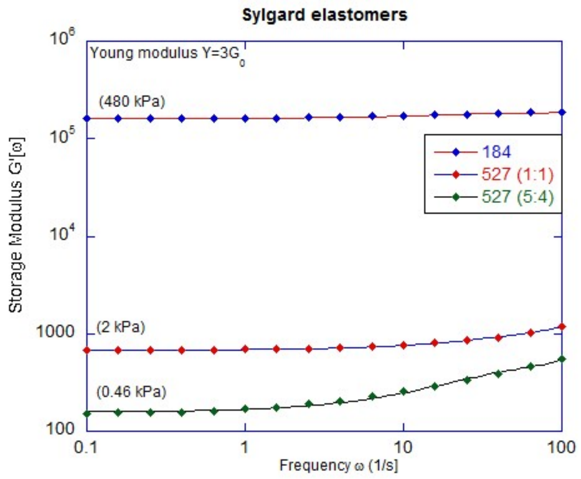

FIG. S1. The results of the rheometry testing of Sylard elastomer substrates: the storage shear modulus $G'(\omega)$ measured in the parallel-plate geometry at constant temperature of 30C. In all cases we are definitely on the low-frequency rubber-modulus plateau. Three materials are displayed: the standard Sylgard 184 elastomer, and two versions of Sylgard 527 elastomers, with 1:1 and 5:4 w/w ratio of the compound to hardener. The value of the equilibrium modulus is labelled on each curve.

It is interesting to note that three orders of magnitude in the magntitude of Young modulus are covered between the different Sylgard elastomer samples in Fig. S1, while the modulus of a standard laboratory glass slide is known from separate measurements to be of the order of 30 GPa, i.e. another 4 orders of magnitude higher than in Sylgard 184.

The spreading criterion

Establishing the unambiguous criterion, by which the cells can be labelled as “spreading” or not, is an important step in our analysis. As in several important publications on the individual dynamics of cell spreading[5–8], we are looking at the point in time, for each individual cell, when its shape crosses over the shape with a contact angle $\sim 90°$. Practically, at this point the bright-field microscope image stops having the ‘lensing effect’ of focusing the illuminating light in the center of the cell image, which for us was the most distinct criterion in the population analysis, Fig. S2.

The interested reader should also check the paper by Frisch and Thoimine[9], where their study of the dynamics of individual cell spreading passes through this precise stage: when the contact angle changes from a large value (above 90°, indicating partial dewetting) to a small value (below 90°, indicating partial wetting). They also quote this transition as the region where the cell cortical tension is approximately mathced by the cell adhesion energy (both of the order $2-4\cdot10^4\mathrm{J/m}^2$).

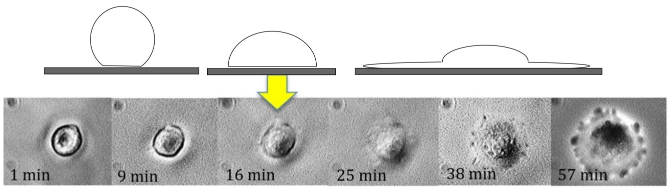

FIG. S2. The illustration of the visual criterion used to count the cells as ‘engaged’ in mechanosensing response. We interpreted the loss of the sharp edge in the microscopic image, and the simultaneous loss of the ‘lensing effect’ with the cell shape becoming flatter than semi-sphere. This point in time was associated with the given cell beginning to spread, and therefore counted towards the ‘fraction of cells engaged in spreading’ in the population.

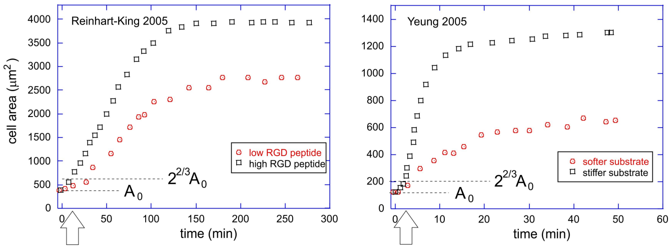

FIG. S3. Plotting some digitized data from Reinhart-King 2005 (fig.2a), and from Yeung 2005 (fig.5d) showing the time evolution of spreading cell area: in the first case at varying concentration of ligands for integrin binding, in the second case at varying substrate stiffness. The initial cell area (on first deposition on surface) is A_0, and our ‘selection criterion’ illustrated in Fig. S2 implies the observed cell area increases by a factor $2^{2/3}\approx1.6$. This is a very early stage of cell spreading, which we refer to as the onset of spreading response.

In order to maximize the sampling size, in our main experiments we have chosen a low magnification and broad field of view, to include around 100 individual cells on the substrate, without contact with each other. At low magnification, the lensing effect is quite pronounced, and so we were able to count the number of cells engaged in spreading in each time-lapsed image without moving the sample.

Quality and reproducibility of data

Our raw data comes in the form of a ‘spreading fraction’, which counts the number of cells in each experiment (with a total in the range of ca. 100 cells in a constant field of view each time), which have passed the spreading criterion at each given time. In order to be able to subject this data to rigorous quantitative analysis, we must be sure it is reproducible, and have a good assessment of uncertainty in each value of ‘spreading fraction’. To this end, we have performed many dozens of separate experiments to assure the reproducibility of their results. For each individual spreading curve (for a given temperature, cell type, substrate, and other conditions) the error bars were quite small – we decided not to include them in the key plots in the main text (Figs. 2 and 3) because they would obscure the distiction between different curves, which was the main point in those plotes.

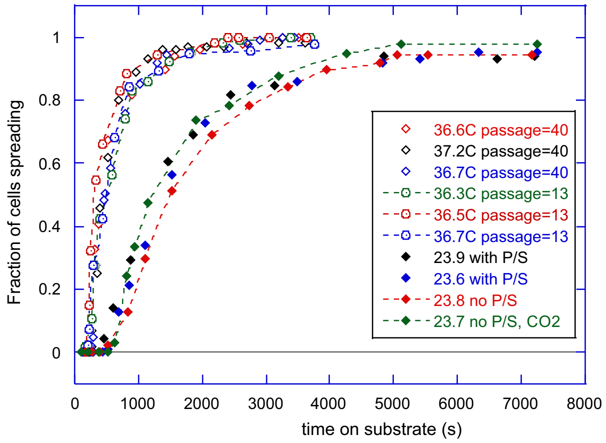

FIG. S4. Comparing the results of a spreading experiment for 3T3 cells of very different passage number, with and without Penn-Strep, and without CO2 tent (all constituting small variations of experimental conditions). The temperature labelled in the plot was the constant actual temperature during the experiment. The error bars, statistically calculated for each data set would be about a half of the spread in the plot.

Here we give an illustration of the robustness of our quantitative data by showing several comparisons. Figure S4 shows the spreading curves obtained in ten separate experiments on 3T3 cells on glass/fibronectin. There are two groups of data (and curves) at two temperatures, aiming to be 37C and 24C. Our device was able to main-

tain the constant temperature during the whole experiment duration due to an active feedback heater system – however, the precision in setting the temperature in advance of each experiment was not greater then $\pm 1°$, hence a spread in actual T-values. We did not regard this variation exceeding the other experimental uncertainties, and in the main text only referred to temperatures with two significant figures.

At the high temperature of 37C, the difference between data sets was in the 'age' of cells: the {young} kind refers to 3T3 cells with the passage number 13, compared to the {old} cells with the passage number 40. Clearly, within this typical range of practical usefullness of reproducing fibroblast cells there data is reproducible. At the lower temperature of 24C we show experiments where we changed the medium environment: with and without Pen/Strep antibiotic, and with a slightly different pH due to removing the standard CO2 tent. In all cases, in spite of such a variation of experimental parameters, the spreading curves are clearly reproducible, and their random variation is not high. The conclusions were the same for EA cells. Therefore, we are assured that the cell counting using our 'spreading criterion' was robust, and fitting of these curves to the model equations reliable.

Fitting data for the long- and short-time relaxation

At long times in our population dynamics we have fitted the single-exponential law of relaxation to saturation, in which all cells are declared 'spreading'. In each case using a fixed offset of the time origin by the 'lag time' (see Fig.3 of the main text), using the equation $f(t) = A \cdot (1 - \exp[-(t - t_{\rm lag})/\tau])$ in two stages: first setting the amplitufe A manually from the apparent saturation level, and using the two-parameter fit to obtain $t_{\rm lag}$ and τ, then fixing $t_{\rm lag}$ and using the two-parameter fit to obtain the saturation amplitude A and the relaxation time τ, which are listed in the table S1. It has turned out that this two-step fitting produced the values of A not differing by more than 5% from the ones we initially set manually, which reassures us in the high quality of this fitting, and the resulting Arrhenius analysis of thermally-activated rate-limiting relaxation presented in Fig. 4 of the main text.

In contrast, in the detailed analysis of initial stages of the cumulative spreading curves in our population dynamics, when only a few cells are beginning to spread (the short-time onset of spreading fraction, see Fig. 5 of the main text), we have discovered that there is no such thing as a 'lag' in this process. The time-resolution of our experiment was naturally limited at very short times: we could not start taking images sooner than 2 min after their planting (since the original culture had to be replaced with the clean medium). Nevertheless, the logarithmic time-axis in Fig.5 of the main text allows a clear view of the power-law regime. We found that the fraction of cells engaged in their spreading process was fitted to the power-law with the fixed power t^5. This has

T(C)	3T3: τ[s]	Max: A	T(C)	EA: τ[s]	Max: A
36.8	275 ± 20	0.99	37	310 ± 21	1.00
36.7	298 ± 19	0.99	36.9	363 ± 27	0.99
36.6	290 ± 20	0.99	36.8	421 ± 23	1.00
36.6	306 ± 18	1.00	36.3	271 ± 21	0.99
36.5	465 ± 22	0.98	35.3	461 ± 33	0.97
36.3	435 ± 35	0.96	29.5	873 ± 58	0.96
35.1	333 ± 28	1.00	27.2	1141 ± 23	0.96
30.2	716 ± 59	0.98	25.4	1201 ± 357	0.81
27.5	1051 ± 82	0.95	25	1282 ± 212	0.81
27.4	804 ± 77	0.99	24.9	1348 ± 228	0.79
26.1	891 ± 82	0.98	24.8	2023 ± 329	0.89
23.9	1122 ± 98	1.00			
23.9	1452 ± 110	0.96			
23.8	1205 ± 103	0.94			
23.8	1815 ± 128	0.98			
23.7	1252 ± 112	0.95			
23.6	1655 ± 143	0.92			

TABLE S1. Values of the parameters of the fitted equation $Q(t) = A(1 - \exp[-t/\tau])$, for different temperatures, and two studied cell types. These values of $\tau(T)$ were used to produce the Arrhenius plot in Fig. 4 in the main text.

T(C)	3T3: α	T(C)	EA: α
36.8	1.51e-13	37	4.93e-14
36.7	1.19e-13	36.9	1.79e-14
36.6	1.34e-13	36.8	4.56e-14
36.6	1.14e-13	36.3	1.44e-14
36.5	8.04e-14	35.3	6.65e-15
36.3	1.03e-13	29.5	3.65e-16
35.1	3.00e-14	27.2	1.07e-16
30.2	5.90e-15	25.4	1.75e-18
27.5	3.73e-15	25	2.63e-18
27.4	3.32e-15	24.9	8.05e-18
26.1	4.28e-15	24.8	5.50e-18
23.9	9.10e-16		
23.9	1.36e-15		
23.8	7.59e-16		
23.8	1.74e-15		
23.7	7.20e-16		
23.6	4.75e-16		

TABLE S2. Values of the prefactor in the universal power-law fitting of short-time data: $Q(t) = \alpha\, t^5$ (for the time taken in seconds), for different temperatures, and two studied cell types. These values of $\alpha(T)$ were used to produce the Arrhenius plot in Fig. 6 in the main text.

been done in two stages: we first fitted the data to an arbitrary power-law αt^{β}, with two fitting parameters α and β, establishing that the exponent β is always close to five. Then we enforced the strict $\beta = 5$ condition, and obtained a fit with just one free parameter α, changing with temperature, which is listed in the table S2.

FAK as reversible mechanosensor

To probe the mechanical modulus of a medium, a force has to be applied to it, either as a local point source, or as distributed stress. The source of this force is the actin-myosin activity of the cytoskeleton delivered via the ATP-rich barbed terminus of F-actin. We need to trace the series of connected devices, from the point of force origin (F-actin) to the point of its application to the ECM. Figure 7 in the main text illustrates this force chain along the assembled protein complex, which has been reproduced in a large number of important publications in this field[10,11].

The integrin family of transmembrane proteins link the extracellular matrix (ECM) to the intracellular actin cytoskeleton via a variety of protein-tyrosine kinases, one of which is the focal adhesion kinase (FAK). Integrins are aggregated in focal adhesions, but at the early stages of activation of cell adhesion mechanisms the focal adhesions are not yet formed. Activation of integrins is required for binding to the ECM proteins: active integrins acquire ligand affinity. It is well established that integrin activation and clustering leads to FAK activation and the subsequent signaling chain of mechanosensing and cytoskeletal remodeling, e.g. see the review by Parsons[12]. There is a large body of literature on integrins, with definitive reviews by Hynes[13,14] explicitly stating that integrins are the mechanosensors. It has recently been demonstrated that the integrin bond with fibronectin has catch-bond characteristics[15], and therefore could have a graded response to force and stiffness.

However, activated integrins possess no catalytic activity, and so cannot act as a mechanosensor on their own. A good summary by Giancotti[16], while talking about integrin signalling, in fact shows schemes where FAK is the nearest to cytoskeletal actin filaments. The important work by Guan et al.[17] establishe a clear correlation chain of extracellular fibronectin transmembrane integrins intracellular FAK, but offers no reason to assume that integrin is the sensing device on this chain. There is a clear indication that phosphorylation of FAK is a key step in the mechanosensing process, e.g. see the review by Geiger et al.[18], pointing at FAK as a fulcrum of this tapestry. Indeed, Schaller et al.[19] state that FAK phosphorylation is the initial step of signaling, and show evidence that crosslinking integrins and ECM (i.e. making the 'substrate' stiffer) leads to an enhanced FAK phosphorylation, while conversely, a damage to integrin is connected with a reduced activation of FAK.

The application of cytoskeletal tension in mechanosensing at focal adhesions is now well established[11]. A key role in this system is played by talin. There are many papers investigating the correlation of talin (as well as paxillin) with β-integrin and FAK; recent studies clearly show that talin is capable of high stretching by a tensile force[20], implying a function similar to that of titin in muscle cells: acting as an extension-limiter. It is also now clear that the immobile domain at the N-terminal of talin is associated with integrin, and also closely associated with the FERM domain of FAK[15], while the C-terminal of talin is associated with paxillin, which in turn may associate with the focal adhesion targeting (FAT) domain (C-terminal) of FAK. Both talin and paxillin also bind to cytoskeletal F-actin. These actin filaments exert a pulling force on the C-terminal of talin, making it play a role of a scaffold for other proteins to arrange around. More importantly, this allows the pulling force to be transmitted from the cytoskeleton to the ECM. This could be used to effect the conformational change in FAK required for its activation. In this model, integrin is merely the bridging element from FAK to the ECM, with the FERM domain localized near the cell membrane and N-terminal of talin. At the opposite end, the FAT domain can be pulled away by the cytoskeletal force transmitted through paxillin/talin. This model is supported by the recent computational study[21], showing that the closed and the open states of FAK are reversibly reached by increasing and decreasing of pulling force.

In our recent theoretical work[22], using this idea of FAK conformational change under applied force, we demonstrated that sensing of stiffness may be a distinct single-molecule response, and develop a theoretical model of reversible mechanosensor. The underlying physics of our model is applicable to a wide variety of protein complexes, but we concentrate on FAK as it occupies a central point in mechanosensing pathways of focal adhesions. The activation of FAK is dependent on cytoskeletal tension, and on ECM stiffness, while the integrin (along with other members of the force chain) is merely playing a role of force transducer. Of course, without the activated integrin there would be no force transduction to ECM, and no mechanosensing. We do not consider the role of clustering into focal adhesions for the early stages of adhesion mechanisms activation.

Nucleation theory leading to power law scaling

At short times, all of our curves exhibit a characteristic power-law dependence on time remarkably, with the same exponent for both types of cells and at all temperatures. In the literature on various kinetic processes, this early-time region is often mistakenly called the 'lag time'; it is clear that there is no such thing as lag just a steep power-law with a possibly high exponent achieves this apparent delay of the growth phase.

Historically, the first time such a process was ana-

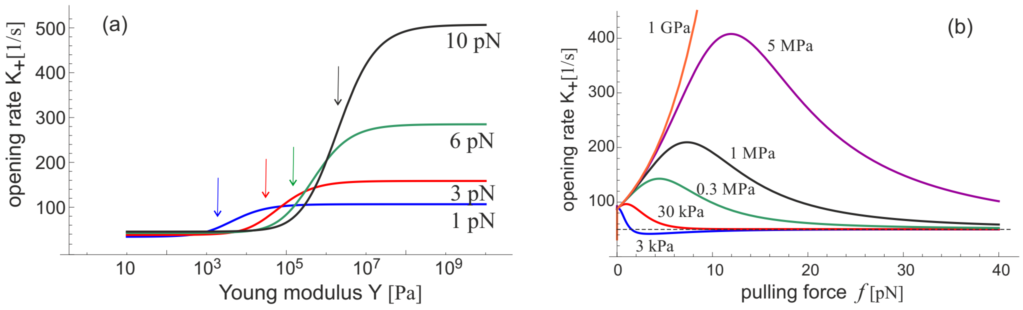

FIG. S5. (a) The rate constant of the FAK activation transition $K_+(f, \kappa)$ plotted against the substrate stiffness (on logarithmic scale) for several values of pulling force f. The arrows point at the inflection point on each curve, i.e. the region of maximum sensitivity. (b) The rate constant $K_+(f, \kappa)$ is plotted as a function of the cytoskeletal pulling force f, for several values of substrate stiffness labelled on the plot. The homeostatic peaks of activation rate $K_+(f)$, for each given substrate stiffness, roughly correspond to the peak of sensitivity in plot (a) at the same level of force. This suggests that the cell self-adjusts the sensor to keep it at the optimal sensitivity on each substrate.

lyzed, and the power-law theoretically reproduced, was in the context of protein self-assembly (gelation of deoxyhemoglobin) by Eaton et al.[23]. They have considered the critical nucleation process, when the aggregating monomers need to reach a nucleus size n_c by adding new particles against an energy barrier while when the size of an aggregate exceeds n_c, the fast polymerization ensues. By assuming the growth of the critical nucleus via a single monomer addition with the same forward rate k_f, it is easy to show by iterative integration that the concentration of the aggregate of N monomers grows at short times as:

$$c_N \approx c_1 \frac{(k_f t)^{n_c - 1}}{(n_c - 1)!}, \tag{1}$$

where c_1 is the initial concentration of monomers. At longer times, additional terms become important, and the rate of growth slows down and saturates when the process is concentration-limited. This analysis is, in turn, similar to the one used by Abraham in the study of kinetics for the nonsteady-state nucleation of supersaturated water vapor. The simple conclusion is: if the kinetics requires an initial assembly of a nucleus made of n_c particles the early time dependence will be the power law with the exponent $(n_c - 1)$, one power per each monomer addition reaction, and the prefactor proportional to the product of rates of individual monomer addition to the growing nucleus.

In fact, the above example of nucleation rate is a particular case of a more general problem in the network theory. Let there be a set of discrete states $\{S_i\}$. These can be represented as nodes of the network, and the transition processes that link these states can be represented as directed edges between these nodes. The process taking the cell from state S_i to state S_j proceeds with a rate k_{ij}. Such a network may have a complex topology, which does not affect the core result we are leading towards. Defining the transition rate matrix of the network, $(\mathbf{Q})ij = k_{ij}$, the probability for the whole process can be written as a vector, $\mathbf{P}(t)$, with each component corresponding to a state Si at time t. Using the Markov chain theory, one can write down the time-dependence of $\mathbf{P}(t)$:

$$\frac{d\mathbf{P}}{dt} = \mathbf{Q} \cdot \mathbf{P} \tag{2}$$

This is called the backwards Kolmogorov equation. If we let state S_0 be the initial state of the process, then the initial condition, on the probability $\mathbf{P}$ at time $t = 0$, is that the first element, corresponding to the probability of being in S_0, is equal to one, and all other elements are zero.

It turns out that at short times, the probability of reaching the final state S_f depends on the length of the shortest path of transitions between S_0 and S_f. A path is defined as the sequence of states the cell passes through as it evolves in time. The probability that the cell will reach the final state, $f(t)$, is given by, at short times:

$$f(t) \approx \frac{k_{01}k_{12}k_{23}...k_{m,f}}{m!} t^m \tag{3}$$

where we label the intermediate states in the process: $[S_0] \to S_1, S_2, , S_m \to [S_f]$, such that the k_{ij} are the rates of the intermediate processes[24]. This is the probability of first passage time at small times, but our experimental data gives the cumulative probability of having reached the final state:

$$P(t) = \int_0^t f(t')\mathrm{d}t' \approx \frac{k_{01}k_{12}k_{23}...k_{m,f}}{(m+1)!} t^{m+1} \tag{4}$$

for short times. Thus, the cumulative probability distribution shows power-law behavior at short times, with the exponent being the minimum number of thermally activated transition processes (the shortest transition pathway) necessary to get from the initial state to final state. At long times, conversely, the first passage time distribution is dominated by the longest timescale in the system (i.e. the system will bottleneck at the slowest transition process). Then, the cumulative probability reverts

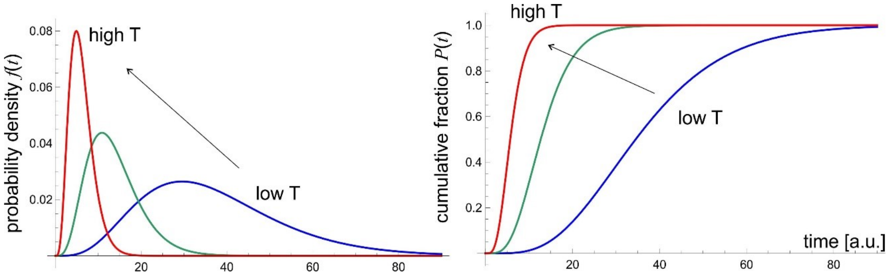

FIG. S6. The illustrations of theoretically predicted probability density $f(t)$ and cumulative fraction $Q(t)$ of mechanosensing activation, based on the crude joining together of the short- and long-time results: the $f(t) = \text{const} \cdot t^4 e^{-t/\tau}$, while the cumulative fraction reproduces the observation data very well.

to the typical two-state single exponential decay as we see in Fig. 4 in the main text.

The critical nucleation kinetics is obviously a very simple example of the general theory, with the sequential addition of monomers to the growing nucleus being the only transition pathway with $m+1 = (n_c - 1)$ steps. Examining the adhesion-mechanosensing complex in Fig. 7 of the main text, discussed in much greater detail in[22], we see that in order to complete the chain of transmitted force from the cytoskeletal F-actin to the adhesion point in the ECM several proteins have to assemble into the complex: The dimer of α- and β-integrins needs to bind to talin, which is needed to activate the integrin, and also provides the anchor for the FERM domain of focal adhesion kinase; at the other end, paxillin and talin are needed to mediate the binding of FAT domain. Our kinetic analysis suggests that there are exactly $n_c = 6$ key players in this complex. Each of these thermally activated binding events has its own rate k_{ij} , and the analysis suggests that the sum of the five energy barriers is 70 kcal/mol for 3T3 fibroblasts, and 130 kcal/mol for endothelial cells. It makes sense to have the rates of adhesion-mechanosensing complex assembly higher in fibroblasts, which have a much more prominent mechanical function. It is possible that this is achieved by an additional companion protein assisting one or several of these binding events.

The main experimental data in this work consists of the cumulative curves, which represent the fraction of cells that have engaged in their mechanosensing response at a given time. We now know that the early-time section of this data is well represented by a t^5 power law, while the late times reflect the simple exponential decay towards the saturation plateau. Importantly, the local gradient of this cumulative curve has the meaning of probability density for the cell to engage at a given time, and combining the two time-regimes together we can illustrate this probability in Figure SS6 with the probability density $f(t) = \text{const} \cdot t^4 e^{-t/\tau}$, and the cumulative fraction $Q(t) = \int_0^t f(t')\mathrm{d}t'$. The maximum of the probability density marks the time at which the cell mechanosensing activation is most likely to occur, while the mean activation time is given by the usual $\langle t \rangle = \int_0^\infty t f(t)\mathrm{d}t$.

[1] D. Bouïs, G. A. P. Hospers, C. Meijer, G. Molema, and N. H. Mulder, "Endothelium in vitro: a review of human vascular endothelial cell lines for blood vessel-related research," Angiogenesis **4**, 91–102 (2001).
[2] R. I. Freshney, *Culture of Animal Cells* (John Wiley & Sons, Inc., 2010).
[3] I. Kuhlmann, "The prophylactic use of antibiotics in cell culture," Cytotechnology **19**, 95–105 (1995).
[4] C. N. D. Cruickshank and E. J. L. Lowbury, "Effect of antibiotics on tissue cultures of human skin," Brit. Med. J. **2**, 1070–1072 (1952).
[5] H.-G. Döbereiner, B. Dubin-Thaler, G. Giannone, H. S. Xenias, and M. P. Sheetz, "Dynamic phase transitions in cell spreading," Phys. Rev. Lett. **93**, 108105 (2004).
[6] Y. Xiong, P. Rangamani, M.-A. Fardin, A. Lipshtat, B. Dubin-Thaler, O. Rossier, M. P. Sheetz, and I. R., "Mechanisms controlling cell size and shape during isotropic cell spreading," Biophys. J. **98**, 2136–2146 (2010).
[7] J. Li, D. Han, and Y.-P. Zhao, "Kinetic behaviour of the cells touching substrate: the interfacial stiffness guides cell spreading," Sci. Rep. **4**, 3910 (2013).
[8] D. Cuvelier, M. Théry, Y.-S. Chu, S. Dufour, J.-P. Thiéry, M. Bornens, P. Nassoy, and L. Mahadevan, "The universal dynamics of cell spreading," Curr. Biol. **17**, 694–699 (2007).
[9] T. Frisch and O. Thoumine, "Predicting the kinetics of cell spreading," J. Biomech. **35**, 1137–1141 (2002).
[10] V. P. Hytönen and B. Wehrle-Haller, "Mechanosensing in cell–matrix adhesions–converting tension into chemical signals," Exp. Cell Res. **343**, 35–41 (2016).
[11] G. Giannone and M. P. Sheetz, "Substrate rigidity and force define form through tyrosine phosphatase and kinase pathways," Trends Cell Biol. **16**, 213–223 (2006).
[12] J. T. Parsons, "Focal adhesion kinase: the first ten years," J. Cell Sci. **116**, 1409–1416 (2003).
[13] R. O. Hynes, "Integrins: versatility, modulation, and signaling in cell adhesion," Cell **69**, 11–25 (1992).
[14] R. O. Hynes, "Integrins: bidirectional, allosteric signaling machines," Cell **110**, 673–687 (2002).
[15] F. Kong, A. J. García, A. P. Mould, M. J. Humphries, and C. Zhu, "Demonstration of catch bonds between an integrin and its ligand," J. Cell Biol. **185**, 1275–1284 (2009).
[16] F. G. Giancotti, "Complexity and specificity of integrin signalling," Nat. Cell Biol. **2**, E13–E14 (2000).
[17] J.-L. Guan and D. Shalloway, "Regulation of focal adhesion-associated protein tyrosine kinase by both cellular adhesion and oncogenic transformation," Nature **358**, 690–692 (1992).
[18] B. Geiger, J. P. Spatz, and A. D. Bershadsky, "Environmental sensing through focal adhesions," Nat. Rev. Mol. Cell Biol. **10**, 21–33 (2009).
[19] M. D. Schaller, C. A. Borgman, B. S. Cobb, R. R. Vines, A. B. Reynolds, and J. T. Parsons, "pp125-fak, a structurally distinctive protein-tyrosine kinase associated with focal adhesions," Proc. Natl. Acad. Sci. USA **89**, 5192–5196 (1992).

[20]M. Yao, B. T. Goult, H. Chen, P. Cong, M. P. Sheetz, and J. Yan, "Mechanical activation of vinculin binding to talin locks talin in an unfolded conformation," Sci. Rep. **4**, 4610 (2014).
[21]J. Zhou, C. Aponte-Santamaría, S. Sturm, J. T. Bullerjahn, A. Bronowska, and F. Gräter, "Mechanism of focal adhesion kinase mechanosensing," PLoS Comp. Biol. **11**, e1004593 (2015).
[22]S. Bell and E. M. Terentjev, "Focal adhesion kinase: the reversible molecular mechanosensor," Biophys. J. **112**, 2439–2450 (2017).
[23]J. Hofrichter, P. D. Ross, and W. A. Eaton, "Kinetics and mechanism of deoxyhemoglobin s gelation: a new approach to understanding sickle cell disease," Proc. Natl. Acad. Sci. USA **71**, 4864–4868 (1974).
[24]A. Valleriani, X. Li, and A. B. Kolomeisky, "Unveiling the hidden structure of complex stochastic biochemical networks," J. Chem. Phys. **140**, 064101 (2014).
